# Organic and Peptidyl Constituents of Snake Venoms: The Picture Is Vastly More Complex Than We Imagined

**DOI:** 10.3390/toxins10100392

**Published:** 2018-09-26

**Authors:** Alejandro Villar-Briones, Steven D. Aird

**Affiliations:** 1 Division of Research Support, Okinawa Institute of Science and Technology, 1919-1 Tancha, Onna-son, Kunigami-gun, Okinawa 904-0495, Japan; avillar@oist.jp; 2 Division of Faculty Affairs and Ecology and Evolution Unit, Okinawa Institute of Science and Technology, 1919-1 Tancha, Onna-son, Kunigami-gun, Okinawa 904-0495, Japan

**Keywords:** snake venoms, metabolites, peptides, purine nucleosides and bases, neurotransmitters, neuromodulators, guanidinium compounds, carboxylic acids, amines, mono- and disaccharides, amino acids

## Abstract

Small metabolites and peptides in 17 snake venoms (Elapidae, Viperinae, and Crotalinae), were quantified using liquid chromatography-mass spectrometry. Each venom contains >900 metabolites and peptides. Many small organic compounds are present at levels that are probably significant in prey envenomation, given that their known pharmacologies are consistent with snake envenomation strategies. Metabolites included purine nucleosides and their bases, neurotransmitters, neuromodulators, guanidino compounds, carboxylic acids, amines, mono- and disaccharides, and amino acids. Peptides of 2–15 amino acids are also present in significant quantities, particularly in crotaline and viperine venoms. Some constituents are specific to individual taxa, while others are broadly distributed. Some of the latter appear to support high anabolic activity in the gland, rather than having toxic functions. Overall, the most abundant organic metabolite was citric acid, owing to its predominance in viperine and crotaline venoms, where it chelates divalent cations to prevent venom degradation by venom metalloproteases and damage to glandular tissue by phospholipases. However, in terms of their concentrations in individual venoms, adenosine, adenine, were most abundant, owing to their high titers in *Dendroaspis polylepis* venom, although hypoxanthine, guanosine, inosine, and guanine all numbered among the 50 most abundant organic constituents. A purine not previously reported in venoms, ethyl adenosine carboxylate, was discovered in *D. polylepis* venom, where it probably contributes to the profound hypotension caused by this venom. Acetylcholine was present in significant quantities only in this highly excitotoxic venom, while 4-guanidinobutyric acid and 5-guanidino-2-oxopentanoic acid were present in all venoms.

## 1. Introduction

In contrast to insect, arachnid, and anuran venoms, snake venom chemistry is dominated by proteins. As a result, relatively little attention has been paid to small organic constituents thereof. Ganguly and Malkana [1] detected cholesterol and lecithin in cobra venom, and Devi [2] claimed that glycerophosphate was present in cobra, viper, and pitviper venoms. Monosaccharides and free amino acids have also been reported, but no role in envenomation for these non-proteinaceous components has ever been suggested. Bieber [3] published the last thorough review of non-proteinaceous snake venom constituents.

Aird [4] proposed that the purine nucleosides adenosine, inosine, and guanosine, documented in various venoms [5,6,7,8,9,10,11], actually occupy a central position in the envenomation strategies of all venomous snakes, whether as venom constituents, or when released from prey tissues by venom proteins and the biochemical cascade they unleash. Later Aird [12,13] documented the purine levels in both snake and helodermatid venoms, confirming that some ophidian venoms comprise as much as 8.7% nucleosides by mass, exceeding the levels of many proteinaceous toxins and lending further credence to his earlier hypothesis about their strategic importance.

Recently, after deproteinating snake venom samples to serve as controls in another study, we were surprised to discover that the small metabolite content of snake venoms is vastly richer than we imagined. While it might have been reasonable to expect trace levels of a broad array of compounds, due to death and replacement of venom gland cells, many of these compounds are 3–6 orders of magnitude more abundant than such anticipated background levels. Accordingly, we identified and semi-quantified small metabolites and peptides in 17 snake venoms representing the families Elapidae (8), Viperinae (2), and Crotalinae (7). Elapid venoms include three individual samples from *Micrurus surinamensis*, in order to assess levels of individual variation; thus, 15 snake taxa were examined.

The diversity of small metabolites seen in these venoms greatly exceeds what has been reported previously. While many of the more abundant compounds have well-known pharmacologies, some, like adenosine, can exert opposing effects, depending upon their tissue concentrations. Like the proteinaceous constituents, titers of these metabolites vary between individuals and between taxa. Undoubtedly, some of them act synergistically with proteinaceous venom components to achieve rapid immobilization of prey.

## 2. Results and Discussion

### 2.1. Compounds Isolated from Snake Venoms

**Figure 1:**
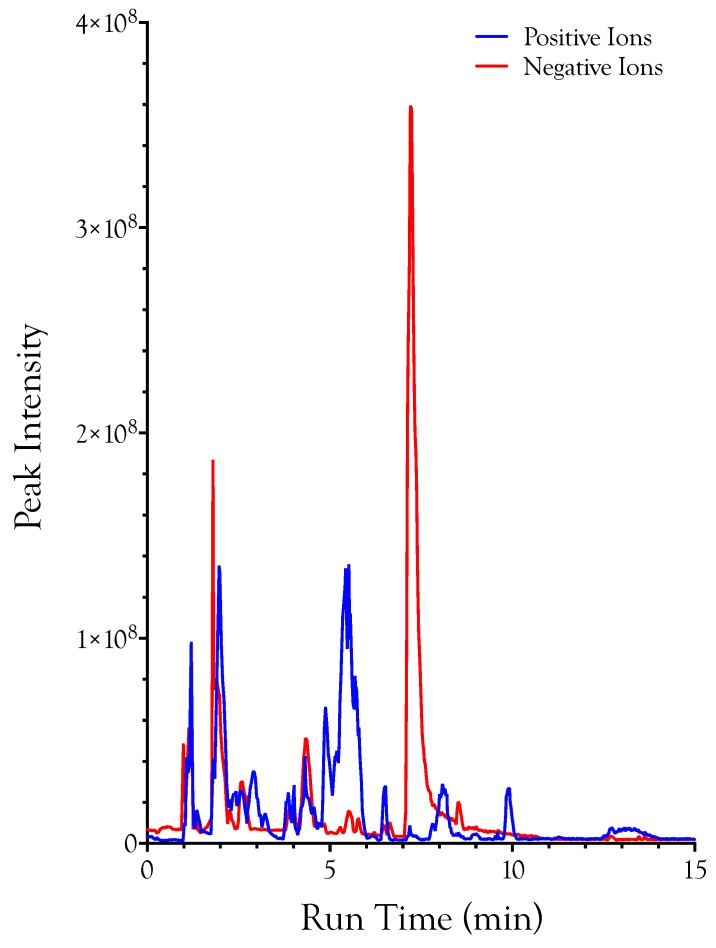
Total ion chromatograms of negative and positive ions of metabolites and peptides from *Agkistrodon piscivorus leucostoma* venom. The negative ion peak that dwarfs all others is citric acid. Assuming no metabolite loss during deproteination, the metabolites and peptides separated here represent the small molecule component of ~69 µg of crude venom. Metabolites were separated on a SeQuant ZIC-pHILIC HPLC 2.1 × 150 mm column, flow rate 120 µL/min, using acetonitrile as solvent A, and 10 mM ammonium carbonate, 0.1% ammonium hydroxide in water as solvent B. Separation was done in HILIC mode, with a linear gradient from 20% to 80% solvent B in 30 min, followed by a wash for 20 min with 20% acetonitrile, 0.5 M sodium chloride in water (solvent C) and, finally, column re-equilibration with starting conditions for 15 min.

Deproteinated samples of 17 snake venoms (Supplementary Table S1) were analyzed by liquid chromatography-mass spectrometry, with simultaneous detection of positive and negative ions (Figure 1). Both positive and negative ions were combined into a master table containing all 17 datasets. Ions were identified on the basis of mass, chromatographic retention time, and fragmentation pattern.

**Figure 2:**
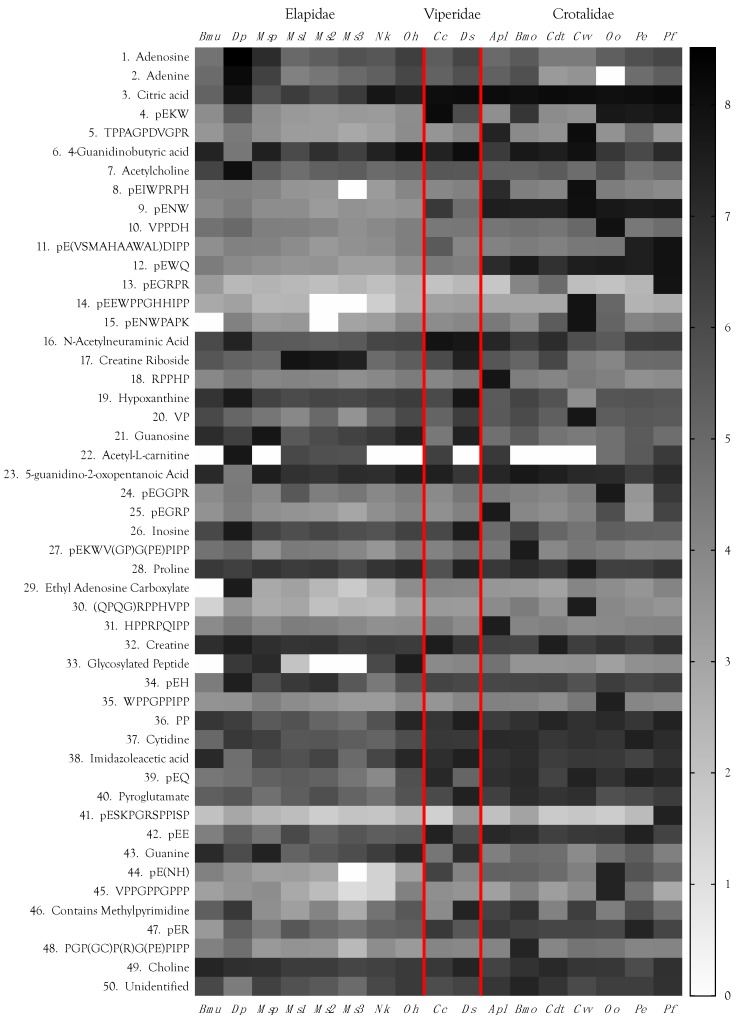
Heat map of the 50 most abundant metabolites and peptides found in 17 snake venoms, arranged in decreasing order of the maximum concentrations found among the species examined. Compound abundances represent the log_10_ of peak intensities of positive and negative ions combined, after subtraction of respective baselines. Logarithmic representations have the effect of compressing apparent differences, so these venoms are compositionally much more divergent than can be shown graphically. Taxonomic names: Bmu, *Bungarus multicinctus*; Dp, *Dendroaspis polylepis*; Msp, *Micrurus spixii*; Ms1–3, *Micrurus surinamensis*, 3 individuals; Nk, *Naja kaouthia*; Oh, *Ophiophagus hannah*; Cc, *Cerastes cerastes*; Ds, *Daboia siamensis*; Apl, *Agkistrodon piscivorus leucostoma*; Bmo, *Bothrops moojeni*; Cdt, *Crotalus durissus terrificus*; Cvv, *Crotalus viridis viridis*; Oo, *Ovophis okinavensis*; Pe, *Protobothrops elegans*; Pf, *Protobothrops flavoviridis*.

Each venom contained roughly 900 LC-MS peaks containing small organic molecules and peptides (<2 kDa). Not all of these peaks represent unique compounds. Some metabolites interact with others during LC, with the result that the same compound occasionally elutes in more than one location. In addition to these duplicate peaks, highly concentrated metabolites, such as adenosine, citric acid, and guanosine, sometimes produced fragments. Still others, such as a couple of tripeptides, were identified not only as monomers, but produced a secondary dimeric peak. Data from these “duplicate peaks” were manually combined. It is safe to say that each venom contained in excess of 850 small organic molecules and peptides. Unquestionably, many more are present at trace levels. For example, we found xanthosine in preliminary experiments, but it was not detected automatically in the final dataset. A manual search in the venom of *Bungarus multicinctus*, where it had previously been most abundant, revealed that it was indeed present, but at levels only slightly above background noise. Accordingly, we have tried to be conservative here. All but one of the 50 most abundant metabolites (Figure 2, Supplementary Table S1) were positively identified, or at least, well characterized. Identifications beyond the top 50 compounds are considered tentative for small metabolites. Peptide sequences provided are confirmed.

Quantities of these small molecules present in each venom differed by orders of magnitude, within and across species, a pattern mimicking that of snake venom proteins [14,15,16] (Figure 2, Supplementary Table S1). Based upon their adjusted areas (the sum of positive and negative ion peak areas for all venoms), the most abundant small venom constituents included carboxylic acids (some of which possessed guanidino groups), purine nucleosides and their bases, neurotransmitters and neuromodulators, amines (mostly tertiary amines), amino acids, and peptides ranging from 2 to 15 amino acids. 

Metabolites identified are discussed below by class and within each class they are presented in order of their decreasing maximum abundance in the venoms examined. That sequence reflects their possible strategic importance in the venoms in which they achieved their highest concentrations, and not necessarily their mean abundance across all venoms examined.

### 2.2. Carboxylic Acids That Chelate Divalent Cations

#### 2.2.1. Citric Acid

**Figure 3:**
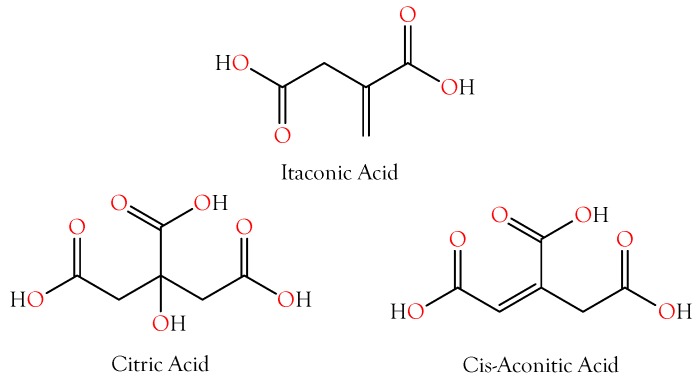
Structures of citric acid, *cis*-aconitic acid, and itaconic acid. *cis*-Aconitate is an intermediate between itaconic acid and citric acid, in the citric acid cycle. It seems probable that itaconic acid and *cis*-aconitic acid exist to support citric acid production in the venom glands. These tricarboxylic acids chelate divalent cations to inactivate phospholipases, metalloproteases, nucleases, and other metalloenzymes in the venom gland; however, upon injection into prey tissues, these components are immediately activated.

**Figure 4:**
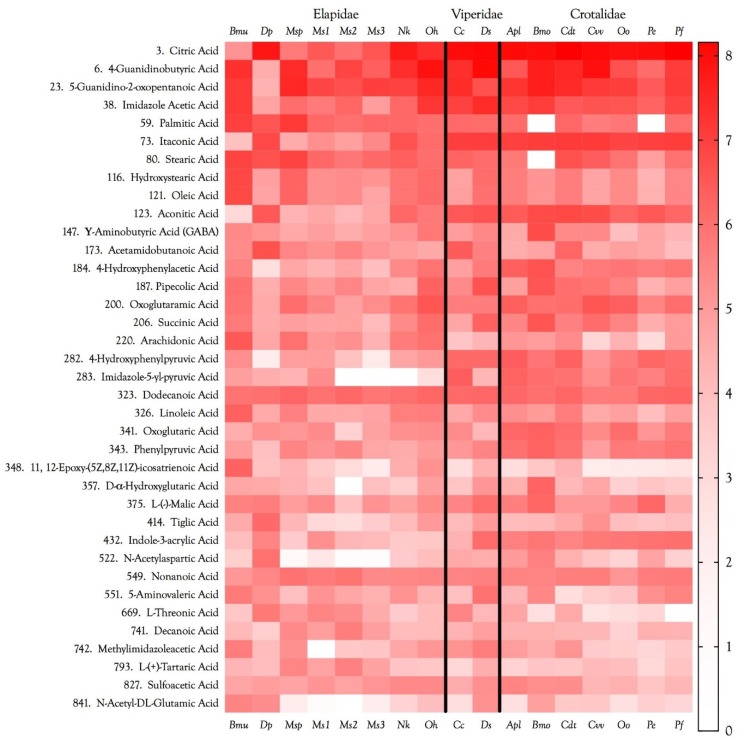
Organic acid abundances in snake venoms span nearly 8 orders of magnitude, based on combined positive and negative ion intensities, after subtraction of the blanks. The vast majority are unquestionably accidental venom constituents, probably resulting from cellular degradation. However, compounds with peak intensities above E^06^, are probably sufficiently concentrated to make substantive contributions to venom pharmacology. Baseline (noise) has been subtracted from all ion intensities. Taxonomic names: Bmu, *Bungarus multicinctus*; Dp, *Dendroaspis polylepis*; Msp, *Micrurus spixii*; Ms1–3, *Micrurus surinamensis*, 3 individuals; Nk, *Naja kaouthia*; Oh, *Ophiophagus hannah*; Cc, *Cerastes cerastes*; Ds, *Daboia siamensis*; Apl, *Agkistrodon piscivorus leucostoma*; Bmo, *Bothrops moojeni*; Cdt, *Crotalus durissus terrificus*; Cvv, *Crotalus viridis viridis*; Oo, *Ovophis okinavensis*; Pe, *Protobothrops elegans*; Pf, *Protobothrops flavoviridis*.

Citric acid was present in in all venoms and was the first-ranked metabolite overall (Figure 3 and Figure 4, Supplementary Table S1). Its concentration was 2–3 orders of magnitude greater in viperine and crotaline venoms than in elapid venoms. Citrate was first discovered in snake venoms in the laboratory of Ivan I. Kaiser [17,18]. Francis et al. [18] found citrate concentrations ranging from 95 to 150 mM in viperine venoms, from 63 to 142 mM in crotalines, and from 17 to 163 mM in elapids. They found that in *Bothrops asper* venom, Ca^2+^ concentrations ranged from 2.5 to 3.6 mM. At those Ca^2+^ concentrations, a phospholipase A_2_ from *Bothrops asper* venom was completely inhibited by only 20 mM citrate. They further reported that *Crotalus adamanteus* 5′-nucleotidase and phosphodiesterase were inhibited 100% and 75%, respectively, by 100 mM citrate [18]. They suggested that citrate may inactivate metalloenzymes in the venom gland by chelating essential metal cofactors. Both phosphodiesterase and 5′-nucleotidase are Mg^2+^-dependent enzymes. It appears that citrate chelates Ca^2+^ more effectively than Mg^2+^, a possibility also implied by Maguire and Cowan [19], who note that a 10× excess of EGTA over Ca^2+^ in a given system would also chelate 20% of the Mg^2+^. Nonetheless, citrate does chelate Mg^2+^ effectively, and many bacterial citrate transporters preferentially transport its Mg^2+^ salt [20].

Odell et al. [21] reported citrate concentrations ranging from 42 to 154 mM (3.6–12.9%) in various elapid, viperine, and crotaline venoms, including a value of 10.3% in *Dendroaspis polylepis* venom. They also found that protease activity of *Crotalus atrox* venom against hide powder azure and azocasein was inhibited 7.5%, and that of *Bothrops picadoi* venom was inhibited 78% with the addition of 18–27 mM exogenous citrate. Thus, the citrate concentrations occurring in venoms should be more than ample to inactivate metalloenzymes, especially considering that venom serine proteases, which do not require metal cofactors, would not have been affected by this treatment. While citrate serves primarily to protect the venom gland from metalloenzymes, given its high concentration in various venoms documented in the foregoing studies and in the present one, it is likely that citrate also functions in envenomation as an anticoagulant, by scavenging Ca^2+^ required by coagulation factors [22,23] and for platelet aggregation [24,25].

#### 2.2.2. Itaconic and *cis*-Aconitic Acids

Itaconic acid was also quite abundant in most venoms, reaching its highest concentrations in viperine and crotaline venoms (Figure 3 and Figure 4, Supplementary Table S1). Like citric acid, for which it serves as a precursor via *cis*-aconitate, itaconic acid is also an excellent chelator of alkali and alkaline earth metals, with divalent cations being bound more strongly than monovalent cations. Using a methylene succinic linker, itaconic acid has been used with a polymeric matrix to make chromatographic columns capable of resolving even very similar ion pairs, such as Ca^2+^ with Sr^2+^ or Mn^2+^ [26]. The literature has nothing to say about the metal chelation capacity of *cis*-aconitate, but it is probably similar to that of citric acid. Venom itaconate levels are only slightly more highly correlated with citrate levels (*r*^2^ = 0.9965) than with *cis*-aconitate (*r*^2^ = 0.9706). *cis*-Aconitate is also highly correlated with citrate (*r*^2^ = 0.9663). The high titers of venom citrate suggest that in venom gland cells, C5-branched dibasic acid and citric acid pathways are being used in unusual ways. Itaconic acid is converted to *cis*-aconitate by aconitate decarboxylase (EC 4.1.1.6). Normally, in the citric acid cycle, aconitate hydratase (EC 4.2.1.3) catalyzes both the conversion of citrate to *cis*-aconitate and *cis*-aconitate to isocitrate. It is not clear how the backward reaction could be promoted and the forward reaction inhibited. However, the mass spectrometer cannot distinguish between citrate and isocitrate, so perhaps much of the citrate is actually isocitrate. From the snake’s standpoint, this probably makes no functional difference, as long as the subsequent step in the citric acid cycle, the conversion of isocitrate to oxalosuccinate by isocitrate dehydrogenase (EC 1.1.1.42), is blocked.

### 2.3. Other Carboxylic Acids

In all, 37 organic acids were identified in the venoms we surveyed. Many more are undoubtedly present, although probably at levels irrelevant to envenomation. Various fatty acids feature prominently among these. However, other organic acids may serve toxic functions in the prey, rather than protective or housekeeping functions in the venom gland. Nearly six decades ago, Curtis and Watkins [27] demonstrated that various carboxylic acids and their α-decarboxylation products have opposing actions on spinal neurons (Table 1), with the former being excitatory and the latter being inhibitory.

#### 2.3.1. 4-Guanidinobutyric Acid

**Figure 5:**
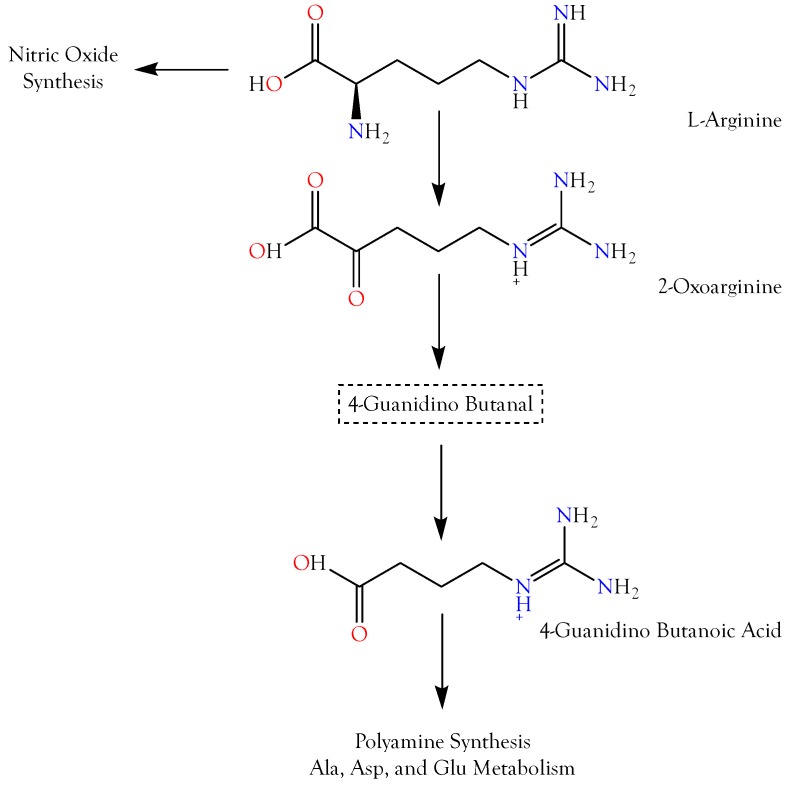
The guanidino group of L-arginine is utilized in the production of nitric oxide (NO). However, l-arginine can also be oxidized to 2-oxoarginine (2OA), a potent convulsant that exerts its effects by blocking chloride channels of GABA_A_ and glycine receptors. With two additional enzymatic reactions, 2OA can be converted to γ-guanidinobutyric acid, which is also a convulsant. Some guanidino compounds also reduce blood pressure and suppress “fight or flight” responses in rats and aggressive behavior in cats. All of these pharmacological effects are consistent with snake envenomation strategies [4].

Three reactions are required to convert l-arginine to 4-guanidinobutyric acid (4GBA), also known as 4-guanidinobutanoic acid (Supplementary Table S1, ID 6) and γ-guanidinobutyric acid (Figure 5); thus, the latter is only two enzymatic reactions removed from 5-guanidino-2-oxopentanoic acid (5G2OA). Overall, this was the second most abundant organic acid (Figure 4 and Figure 5). Compounds with imidazole or guanidinium groups inhibit the vasodilatory actions of K_ATP_ channel openers [28], but since a hypertensive function would make little sense in envenomation by most snakes, like 5G2OA, the pharmacological function of 4GBA is probably its capacity to induce seizures. Hiramatsu [29] and Tachikawa and Hosoya [30] reported that accumulation of guanidino compounds in the brain may induce epileptic discharges and convulsions. Jinnai et al. [31] found that cisternal injection of rabbits with 5 mg/kg of 4GBA caused both tonic and clonic seizures, although intravenous injection of 5 or 25 mg/kg of 4GBA did not. The epileptogenicity of guanidino compounds apparently stems from their inhibition of the inhibitory actions of GABA_A_ and, possibly also, glycine receptors [32,33]. The failure of the nonbenzodiazepine anxiolytic drug, CGS 9896, to reverse the antagonism of GABA activity by guanidino compounds, indicated that they act at a site distinct from the GABA binding site.

Why do snakes employ both 5G2OA and 4GBA? De Deyn et al. [34] found that inhibitory effects of guanidine and methylguanidine on GABA were additive, and it is possible that 5G2OA and 4GBA also act synergistically, likely by stimulating distinct sites on the GABAR (i.e., on the Cl^−^ channel and on an allosteric site), or on different subclasses of GABARs [33,35,36]. Both pharmacological activities are capable of inducing epileptic seizures by reducing GABAergic inhibition in the central nervous system [37,38,39].

The superior cervical ganglion is a part of the autonomic nervous system and is specifically responsible for “fight-or-flight” responses. Antagonism of GABAergic neurotransmission in the posterior hypothalamus elicits escape locomotor behavior in rats [40], whereas stimulation of GABAergic signaling suppresses such behavior, which would be to the advantage of the snake. Kása et al. [41] and Wolff et al. [42] found that GABAergic axons are distributed unevenly within the superior cervical ganglion. GABAergic innervation of the soma and the stem dendrites of a subpopulation of principal ganglion cells was especially significant, suggesting that GABAergic innervation is critical to the function of this ganglion. Two enzymes involved in GABA metabolism, glutamate decarboxylase and GABA-transaminase, have also been localized in neurons of sympathetic ganglia [43,44]. Galvan et al. [45] reported that in the rat isolated superior cervical ganglion, in 4-aminopyridine solution (200–300 µM), 100 µM GABA, evoked “bursts” of spikes and EPSPs, in addition to neuronal depolarization.

Relative to envenomation, the importance of 4GBA’s action in the superior cervical ganglion may pertain to its agonism of GABA receptors. 4GBA depolarized rat superior cervical ganglia in the same manner as GABA, but with only about 1% the potency thereof [46]. Siegel and Schubert [47] reported that a GABAergic pathway from medial to lateral hypothalamus suppresses aggressive behavior in cats. Nonetheless, it is unclear whether the quantities of 4GBA detected here would be sufficient to affect the superior cervical ganglia, even if it does act as suggested.

Takahashi et al. [48] reported that iv injections of GABA in anesthetized rabbits produced hypotension and bradycardia. Similar results were obtained with GABA injections into dogs, cats, and rats [49,50,51]. Serafin et al. [52] found that 2-guanidino-benzimidazole and 2-guanidino-5-aminobenzimidazole dihydrochloride had hypotensive activity, comparable to that of aminophylline. Thomas et al. [53] reported that two guanidino compounds, l-homoarginine and l-amino-tau-guanidino butyric acid, behave like l-arginine in reversing the vasoconstrictor effect of l-NMMA, in a stereospecific and concentration-dependent manner. However, l-amino guanidino propionic acid and guanidine were ineffective. Thus, as guanidino compounds, both 5G2OA and 4GBA may be hypotensive, which would be consistent with snake envenomation strategies [4].

4GBA may also have antimicrobial actions in the venom gland, given that 4GBA and other guanidino acids are effective at suppressing bacillus and coccus infections [54]. However, such action would likely be secondary to a role in envenomation.

#### 2.3.2. 5-Guanidino-2-oxopentanoic Acid

5-Guanidino-2-oxopentanoic acid (5G2OA), also known as 2-oxoarginine, is the first metabolite of arginine catabolism (Figure 5). 5G2OA was the third most abundant organic acid among the venoms we surveyed, based upon their maximal concentrations (Supplementary Table S1, ID 22). 5G2OA levels are increased in patients with argininemia, a deficiency of the enzyme arginase (EC 3.5.3.1). Among arginine metabolites, 5G2OA has been especially implicated in the central nervous system damage that occurs in that disease [55]. De Deyn et al. [35,36] first suggested that the convulsant effects of 5G2OA in rabbits might be due to a blockade of chloride channels associated with GABA and glycine receptors, thus inhibiting responses to these inhibitory neurotransmitters. Later, GABA_A_ receptors were specifically implicated [33] in its convulsant effects.

#### 2.3.3. Imidazole-4-acetic Acid

**Figure 6:**
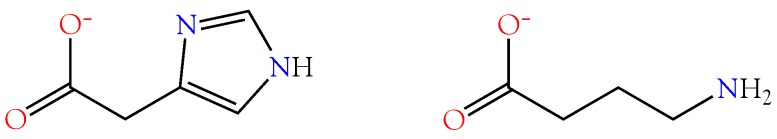
Imidazole-4-acetic acid, (left) an agonist of mammalian GABA_A_ receptors. The natural agonist, γ-amino butyric acid, or GABA, is shown on the right.

Imidazole-4-acetic acid (I4AA) (Figure 6) is the fifth most abundant carboxylic acid occurring in the venoms we tested. Found in the brains of mammals, it is a naturally occurring histidine metabolite that is structurally similar to GABA [56,57,58,59,60,61]. Numerous studies have reported I4AA pharmacology that is consonant with snake envenomation. When applied iontophoretically to cat cortical neurons stimulated with glutamate, I4AA inhibited neuronal firing in a manner similar to GABA [62], but with a slower onset [63]. Roberts and Simonsen [64] found that I4AA had sedative and analgesic effects when injected ip (4 µg/g) into mice. A subsequent study from the same group reported that mice injected with increasing doses from 1 to 3 µmol/g displayed hyperactivity, ataxia, catalepsy and, finally, complete loss of a righting reflex [65]. Similar results were obtained in rats. Tunnicliff et al. [66] discovered that I4AA injected ip into mice at 3 µmol/g caused body temperature to decrease steadily over a 2 h time course. Sooriyamoorthy et al. [67] found that I4AA (500 mg/kg) in conscious rats decreased cerebral blood flow by 42%. 

Roberts [68] reported that I4AA displaces ^3^H-GABA from receptor-related membrane sites with an IC_50_ of 1.3 µM (Figure 6). I4AA acts as an agonist at GABA_A_ receptors [59,69,70], and has been reported as everything from a partial agonist to a strong antagonist at GABA_C_ receptors [56,61,70,71,72,73,74,75]. Binding of the ligand, GABA, to GABA_A_ or GABA_C_ receptors, opens a chloride channel [76]. Whereas GABA_A_ receptors produce rapid, transient responses, GABA_C_ receptors promote rapid, prolonged responses [77]. While GABA_C_ receptors are widely distributed, they are much less abundant than GABA_A_ and GABA_B_ receptors [78], and their pharmacology and response speed do not seem particularly pertinent to envenomation.

On the other hand, GABA_A_ receptors are ionotropic, and occur as heteropentamers [79]. GABA_A_ agonists reduce neuronal excitability and exhibit sedative effects [80]. For instance, the partial GABA_A_ agonist, MRK-409, induces sedation in humans at only ~10% receptor occupancy. I4AA inhibits the firing of CNS neurons [62]. It readily crosses the blood–brain barrier when administered systemically, whereupon it decreases blood pressure and heart rate by agonizing GABA_A_ receptors in the CNS [81]. Both effects accord perfectly with the envenomation strategies identified by Aird [4].

Roberts and Simonsen [82] discovered that I4AA activates 3′,5′-nucleotide phosphodiesterase by binding to an allosteric site. Since this enzyme degrades cAMP to AMP, the latter, which is somewhat hypotensive, can be dephosphorylated to release adenosine, which is strongly so. Thinking that a decrease in cAMP might reduce blood pressure, Walland [83] injected I4AA into the lateral ventricle of the brain in cats, and found that it elicited dose-dependent hypotension.

In vertebrate retina, the taurine transporter (TAUT) is largely responsible for retinal transport of GABA, in contrast to the brain, where the GABA transporter has a larger functional role [84]. Retinal taurine influx is significantly inhibited in a concentration-dependent manner by both GABA and by I4AA [85]. GABA transporter 2 (GAT-2, also known as BGT-1) is also a taurine transporter [86], although its affinity for GABA is ~30× higher than its affinity for taurine [87,88]. It is unclear whether I4AA also inhibits GAT2, although this would not be surprising.

#### 2.3.4. 4-Hydroxyphenylacetic and 4-Hydroxyphenylpyruvic Acids

**Figure 7:**
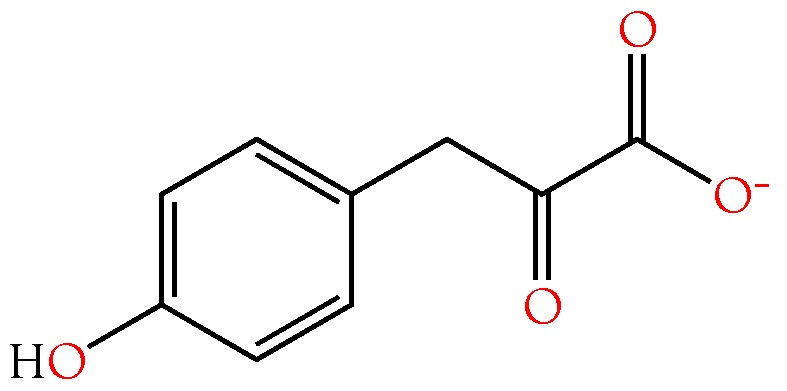
Structure of 4-hydroxyphenylpyruvic acid, an inhibitor of acetylcholinesterase produced by the action of venom l-amino acid oxidase on tyrosine [93].

4-Hydroxyphenylpyruvic acid (4HPPA) (Figure 7), like 4-hydroxyphenylacetic acid (4HPAA), occurs at moderate levels in all crotaline venoms (Figure 4). It is essentially absent from *Dendroaspis polylepis* venom, and is minimal in most other elapid venoms. Very little is known about this compound. The biomedical literature is likewise all but silent on it. However, tyrosine can be catabolized by tyrosine aminotransferase (EC 2.6.1.5) to form 4HPPA and glutamate [89]. 4HPPA, in turn, can be converted to homogentisic acid by the action of 4-hydroxyphenylpyruvate dioxygenase (EC 1.13.11.27). Szwajgier [90] reported that of nine phenolic acids tested, homogentisic acid and 4HPPA were the most effective acetylcholinesterase inhibitors, using the spectrophotometric method of Ellman et al. [91]; however, many phenolic acids are inhibitors of both acetylcholinesterase and butylcholinesterase [92]. This inhibitory activity would be consistent with the mamba envenomation strategy, thus, its complete absence from *D. polylepis* venom is unexpected (Figure 4), raising the question of whether this really is its primary function.

Nucaro et al. [93] reported that in vitro, Taiwan cobra (*Naja atra*) venom is able to convert tyrosine into phenolic oxidation products via 4-hydroxyphenylpyruvate (Figure 7). They found that tyrosine was released from neuropeptides by oligopeptidases in the venom. Nucaro et al. [93] determined that venom l-amino acid oxidase (LAO) initially releases the keto form of 4-hydroxyphenylpyruvic acid and hydrogen peroxide using tyrosine as a substrate. They asserted that, thereafter, a venom tautomerase effects a partial conversion of the keto form of 4HPPA into an enol. The latter is oxidized to 4-hydroxybenzaldehyde and 4-hydroxyphenol, while the keto form is oxidized to 4-hydroxyphenylacetic acid by H_2_O_2_ co-released from tyrosine by LAO. Utilizing both of these oxidation routes, *Naja atra* venom generates still other phenolics [93]. Whether the venom titers of these compounds are sufficient to influence prey envenomation is debatable. Nonetheless, it seems likely that quantities of 4HPPA and 4HPAA released in the prey by venom enzymes may well be significant.

#### 2.3.5. Indole-3-acrylic Acid 

Xanthine oxidase oxidizes hypoxanthine to xanthine for subsequent conversion to uric acid, but degradation of hypoxanthine is blocked by indole-3-acrylic acid (I3AA), which inhibits xanthine oxidase with an IC_50_ of 136 µM [94]. Therefore, indole-3-acrylic acid could potentially contribute to inosine accumulation in the venom gland, or it may block degradation of hypoxanthine to xanthine by prey xanthine oxidase, driving conversion of hypoxanthine to inosine by prey enzymes.

Kynurenine aminotransferase (KAT1) converts kynurenine into kynurenic acid, an inhibitor of NMDA glutamate receptors [95,96,97,98] and α7-nicotinic (neuronal) acetylcholine receptors [96,97,99]. I3AA inhibits human KAT1, thereby blocking formation of kynurenic acid [100]. As a result, the net effect of I3AA on NMDA iGluRs and α7 nAChRs would depend upon the balance between exogenous and endogenous agonists and antagonists, a balance that could differ depending upon the snake involved. Interestingly, mamba (*Dendroaspis*) venoms, arguably the most excitatory snake venoms known, do not contain I3AA. However, this is probably because they already contain dendrotoxins [101,102], fasciculins [103,104,105], and acetylcholine [106]. This combination presumably floods nicotinic synapses with neurotransmitter, causing fasciculations and, also, promotes hypotension via vascular M3 muscarinic AChRs [107,108,109,110]. For a review of muscarinic receptors in snake envenomation, see Aird et al. [16].

**Figure 8:**
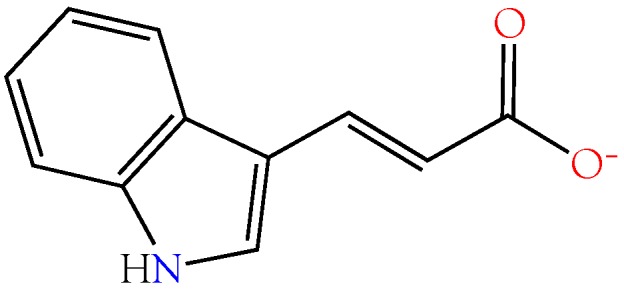
Structure of indole-3-acrylic acid, an inhibitor of xanthine oxidase, kynurenine aminotransferase, and d-dopachrome tautomerase.

**Figure 9:**
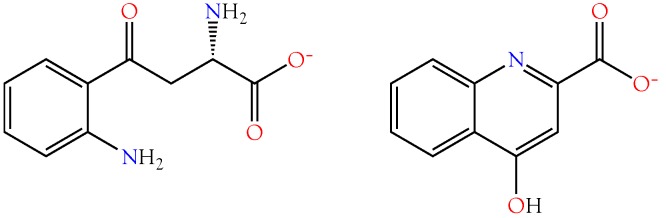
Structures of l-kynurenine (left) and kynurenic acid (right). l-kynurenines such as quinolinic acid are excitatory, but kynurenic acid, produced from l-kynurenine by the action of kynurenine aminotransferase (KCAT1), is an inhibitor of NMDA iGluRs and α7 nAChRs. I3AA inhibits KCAT1, blocking production of both molecules by this pathway.

I3AA (Figure 8) is also a potent inhibitor of mammalian tryptophan 2,3-dioxygenase, although it does not inhibit indoleamine 2,3-dioxygenase, an enzyme that degrades 5-hydroxytryptamine and serotonin [111]. Tryptophan 2,3-dioxygenase deficiency results in neuroprotection because it degrades tryptophan to kynurenine, a precursor to kynurenic acid (Figure 9). Interestingly, the two related compounds have opposing pharmacologies [112]. Kynurenines, such as quinolinic acid are excitatory, while kynurenic acid is inhibitory.

Lastly, I3AA also potently inhibits D-dopachrome tautomerase, an isomer of macrophage migration inhibitory factor (MIF) [113]. Despite its discovery decades ago, the natural substrate of MIF remains unidentified [114]; hence, it is impossible to say exactly what effects its blockade by I3AA might have.

#### 2.3.6. 5-Aminolevulinic Acid

**Figure 10:**
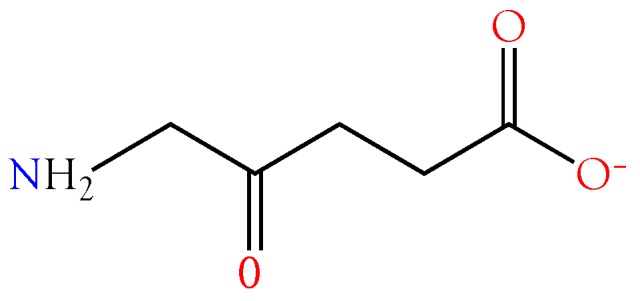
Structure of 5-aminolevulinic acid.

Hermes-Lima [115] reported that 5-aminolevulinic acid (Figure 10) generates oxygen radicals in vitro and, possibly, in vivo during pathologic situations in which its concentration is elevated. The damage is ascribed primarily to –OH radicals. Bechara [116] found that 5-aminolevulinic acid undergoes transition metal-catalyzed oxidation to yield O^−2^, H_2_O_2_, and HO^−^. 5-Aminolevulinic acid was present in all venoms at low to very low concentrations (Figure 4). Oddly, it was most abundant in venoms of *B. multicinctus* and *D. siamensis*.

### 2.4. Purine Nucleosides

#### 2.4.1. Adenosine

Adenosine contributes to prey immobilization by activation of neuronal adenosine A_1_ receptors [117,118,119,120,121,122,123,124], suppressing acetylcholine release from motor neurons [125,126,127,128,129,130] and excitatory neurotransmitters from central sites [131,132,133]. It also exacerbates venom-induced hypotension by activating A_2_ receptors in the vasculature, and by depressing cardiac output and causing atrioventricular block [134,135,136,137,138,139,140,141]. Inosine potentiates the coronary vasodilatory effects of adenosine [142,143]. Aird [144] also reviewed numerous secondary effects of adenosine that are germane to snake envenomation.

**Figure 11:**
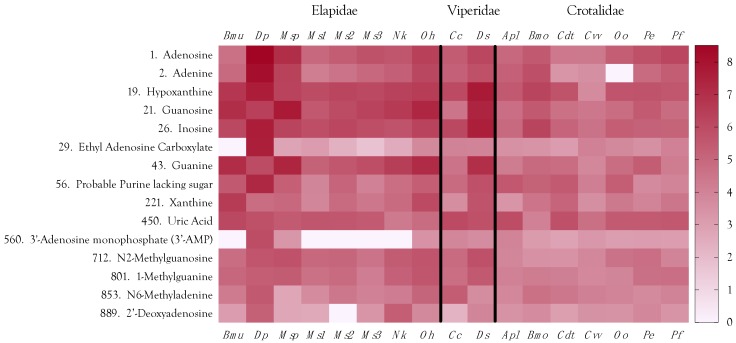
Purine nucleosides and their bases are significant constituents of elapid, viperine, and crotaline venoms. Elapid and viperine venoms contain greater quantities of them than crotaline venoms. The purine strategy of *D. polylepis* is particularly noteworthy. Taxonomic names: Bmu, *Bungarus multicinctus*; Dp, *Dendroaspis polylepis*; MSP, *Micrurus spixii*; Ms1–3, *Micrurus surinamensis*, 3 individuals; Nk, *Naja kaouthia*; Oh, *Ophiophagus hannah*; Cc, *Cerastes cerastes*; Ds, *Daboia siamensis*; Apl, *Agkistrodon piscivorus leucostoma*; Bmo, *Bothrops moojeni*; Cdt, *Crotalus durissus terrificus*; Cvv, *Crotalus viridis viridis*; Oo, *Ovophis okinavensis*; Pe, *Protobothrops elegans*; Pf, *Protobothrops flavoviridis*.

The most abundant small organic compounds overall were adenosine and adenine, owing principally to their extremely high concentrations in *Dendroaspis polylepis* venom, which exceeded the levels found in the remaining 16 venoms by 1–4 orders of magnitude (Figure 2 and Figure 11, Supplementary Table S1). High levels of adenosine have previously been reported for *Dendroaspis angusticeps* venom [12,145]. Aird [4,144] proposed that purine nucleosides (adenosine, inosine, and guanosine) are central to the envenomation strategies of nearly all venomous snakes, either as exogenous components co-injected with proteinaceous toxins, or as endogenous secondary messengers released from prey tissues by the action of enzymatic venom constituents. The first part of that hypothesis has been amply documented [12,145,146,147,148,149], and several recent studies have also provided strong support for the second part.

Cintra-Francischinelli et al. [150] reported that *Bothrops asper* myotoxins release large quantities of ATP and K^+^. Building upon that earlier work, Caccin et al. [151] provided elegant proof that the purified catalytic (Asp49) and non-catalytic (Lys49) phospholipase myotoxins from *Bothrops asper* venom induce rapid release of ATP from mouse skeletal muscle, as predicted. Contrary to their expectations that crude venom would release even more, it did not seem to do so. This apparent contradiction was explained by the fact that the crude venom contains both phosphodiesterase and 5′-nucleotidase, which rapidly degrade ATP to adenosine. They concluded that high concentrations of adenosine are released by the combined myotoxic and enzymatic activities of the venom and that the adenosine contributes to prey immobilization.

Likewise, Tonello et al. [152] found that even at sublytic doses, Mt-II, the non-catalytic myotoxin from *Bothrops asper* venom, induced a dose-dependent release of ATP from mouse macrophages, triggering Ca^2+^ release from intracellular stores that resulted in cell death in less than 1 h. The cell death process appears to involve binding of Mt-II to PX1, 2, or 3 receptors, as well as to PY12 and PY13 receptors, which results in further ATP release [152]. However, the initial release of ATP may be involved in a positive feedback loop that facilitates the second release.

**Figure 12:**
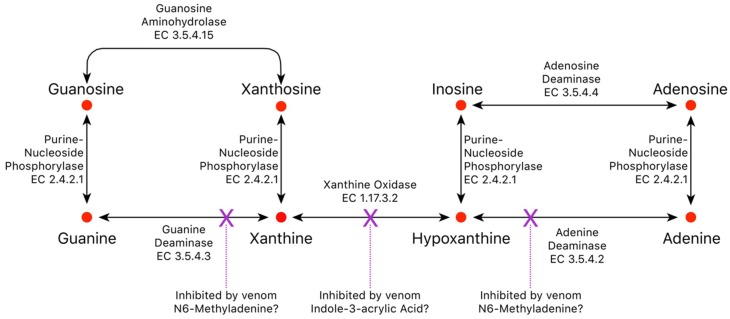
Elapid and viperine venoms contain high levels of purine nucleosides, while crotaline venoms tend to have trace quantities [4,12]. However, crotaline venoms accomplish the same objective by releasing purines from prey tissues. Venom purines isolated during this study are indicated by red dots. Interestingly, blockade of xanthine oxidase by venom indole-3-acrylic acid might drive hypoxanthine toward inosine or adenine in the venom gland; however, it is most abundant in crotaline venoms (Figure 11), which have very low purine titers. Therefore, this function seems unlikely. Perhaps it serves a similar function in prey tissues. Venoms also contain much lower levels still of *N*^6^-methyladenine, an inhibitor of both adenine and guanine deaminases; however, this compound is most abundant in mamba venom, which employs a purinergic envenomation strategy. Thus, it may support adenosine synthesis in some fashion, perhaps by blocking the backward conversion of adenine to hypoxanthine.

**Figure 13:**
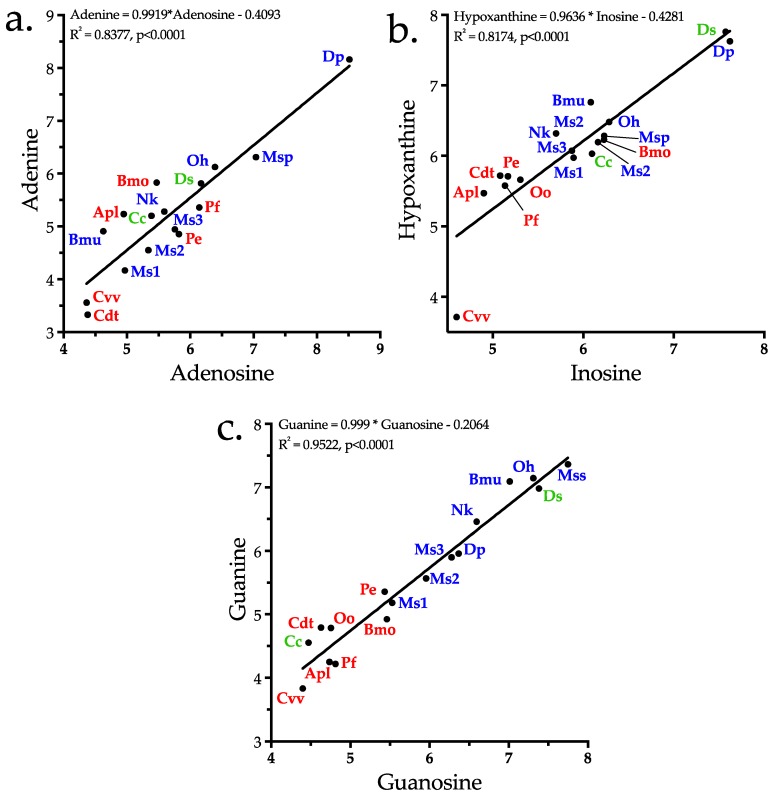
Concentrations of purine bases in venoms are highly correlated with concentrations of their respective nucleosides, suggesting that their primary function is to support production of the nucleosides, the roles in envenomation of which have been well characterized. *Ovophis okinavensis* was excluded from plot 13a because no adenine was detected in that venom. Elapids, red; viperines, green; and crotalines, blue. Taxonomic abbreviations: Apl, *Agkistrodon piscivorus leucostoma*; Bmo, *Bothrops moojeni*; Bmu, *Bungarus multicinctus*; Cc, *Cerastes cerastes*; Cdt, *Crotalus durissus terrificus*; Cvv, *Crotalus viridis viridis*; Ds, *Daboia siamensis*; Dp, *Dendroaspis polylepis*; Mss, *Micrurus spixii spixii*; Ms1–3, *Micrurus surinamensis* 1–3; Nk, *Naja kaouthia*; Oh, *Ophiophagus hannah*; Oo, *Ovophis okinavensis*; Pe, *Protobothrops elegans*; Pf, *Protobothrops flavoviridis*.

In addition to adenosine, other purines, adenine, inosine, hypoxanthine, guanosine, guanine, xanthine, and xanthosine, are also present, with all but xanthine and xanthosine ranking among the top 43 metabolites. While the purine bases, adenine, hypoxanthine, and guanine, may have pharmacological actions that are consonant with ophidian envenomation strategies, we propose that they exist in venoms primarily as substrates for conversion to their corresponding nucleosides by purine-nucleoside phosphorylase (EC 2.4.2.1) (Figure 12). In possible support of this contention, regression analysis showed that concentrations of all three bases are highly correlated with concentrations of their nucleosides for the 17 species examined here (adenine–adenosine, *r*^2^ = 0.444 (*p* = 0.0035); hypoxanthine–inosine, *r*^2^ = 0.8174 (*p <* 0.0001), guanine–guanosine, *r*^2^ = 0.9522, (*p <* 0.0001)) (Figure 13a–c).

#### 2.4.2. Inosine

Inosine potentiates the coronary vasodilatory effects of adenosine [142,143]. Like adenosine, inosine activates mast cell A_3_ receptors, liberating vasoactive substances and increasing vascular permeability [153,154,155,156]. Fuentes et al. [157] reported that like adenosine, inosine inhibited platelet aggregation and ATP release stimulated by ADP and collagen. Both nucleosides significantly prevented thrombus formation in vivo, apparently acting at platelet adenosine A2A receptors. Other reports also suggest that inosine is capable of acting as an A2A receptor agonist [158,159]. Since the half-life of inosine (15 h) in vivo is much longer than that of adenosine (10 s), inosine provides a way of extending the pharmacological action of adenosine at A2A receptors [160]. Inosine has been identified as an endogenous ligand of the benzodiazepine binding site of GABA_A_ receptors [161], suggesting that it may contribute to sedation and ataxia [162] caused by adenosine [163].

#### 2.4.3. Guanosine

Aird [12] noted that guanosine tends to be more abundant in venoms of snakes that prey upon reptiles, amphibians, or fish. That pattern holds for this dataset as well. *Micrurus spixii* presented the highest levels of both guanosine and guanine, followed by *B. multicinctus* and *O. hannah*, *Daboia siamensis* actually had slightly more elevated levels than *O. hannah*, but nothing is known about the prey preferences of this taxon. Interestingly, venom of *P. elegans*, a small habu from the Sakishima Islands in western Okinawa Prefecture that feeds largely on lizards, has much higher levels of guanosine and guanine than the closely related *P. flavoviridis*, from Okinawa Island, which attains lengths of 2.5 m, and which feeds almost entirely on small mammals upon reaching adulthood.

Upon intradermal injection in humans, xanthosine causes pain, but unlike inosine and guanosine, it does not liberate histamine [164]. Hayashi et al. [165] reported that inhibition of neurotransmitter release from guinea pig ileal strips was shifted to the right by xanthine derivatives; thus, xanthosine acts as an adenosine inhibitor, and its known pharmacological activities run contrary to snake envenomation strategies [4]. Xanthosine shows a definite phylogenetic distribution in venoms. While crotaline venoms are essentially devoid of it, xanthosine is variably present in elapid and viperine venoms, but at very low levels. In fact, in our second analysis, xanthosine was not detected, owing to a loss of sensitivity. Given its pharmacology, why would it be present in any snake venoms? The answer appears to be that xanthosine concentration correlates well with guanosine concentration (Figure 13c), suggesting that it exists in venom primarily for conversion to guanosine by guanosine aminohydrolase (E.C. 3.5.4.15) (Figure 12).

#### 2.4.4. Ethyl Adenosine Carboxylate (EAC)

A form of ethyl adenosine carboxylate (EAC) is present in *D. polylepis* venom at very high concentrations (Figure 11). It appears in trace quantities in all other venoms, except that of *B. multicinctus*, which is entirely negative. Fragmentation was inadequate to identify to isomer that occurs in *D. polylepis* venom, and attempts to fragment it further upon re-isolation were not successful. Only one isomer, ethyl adenosine-5′-carboxylate, appears in the biomedical literature.

Imai et al. [166] reported that, in doses >30 µg, EAC produced a pronounced, transient increase in coronary blood flow in dogs, accompanied by slight bradycardia. Adenosine, in doses >1 mg, produced similar effects to those of EAC, except that the bradycardia was more pronounced and of briefer duration. The authors concluded that EAC exerted a direct vasodilatory effect on the coronary vasculature. EAC’s effects were antagonized by aminophylline in the same fashion as those of adenosine [166]. Moreover, those effects were not potentiated by dipyridamole, an adenosine potentiator. EAC’s pharmacology would be entirely consistent with the *Dendroaspis* purine-based, hypotensive, envenomation strategy [4].

#### 2.4.5. Minor Purines

After guanine, the next most abundant purine was an unidentified compound that is most likely a non-standard purine base derivative, devoid of sugar (Figure 11). It is quite abundant in *D. polylepis* venom, suggesting that it is no artefact. Unfortunately, this compound underwent very little fragmentation, thwarting attempts to identify it.

1-Methylguanine, 7-methylguanosine, and *N*^6^-methyladenine were also found in various venoms, but at concentrations 2–5 orders of magnitude lower than the concentrations of EAC found in *D. polylepis* venom (Figure 11, Supplementary Table S1). 1-Methylguanine and 7-methylguanine, metabolic products of tRNA degradation, induced a 50% increase of Con A-mediated hemadsorption within 20 h of exposure of the cells to the agent at a concentration of 10^−5^ M [167,168]. It may be that these compounds promote platelet aggregation, or stimulate mast cells, since they affect membrane characteristic of blood cells. However, they are present in very small quantities, and may not have a significant effect on any physiological parameter in the prey; however, titers are higher in elapid and viperine venoms than in crotaline venoms. The 1- and 7-methyl isomers cannot be distinguished readily by mass spectrometry.

*N*^6^-Methyladenine is present in most, if not all, invertebrates and vertebrates at very low levels. Evolutionarily, it is a highly conserved epigenetic marker that governs gene expression [169]. It is present in snake venoms (Figure 11) at levels that may approach housekeeping levels; nonetheless, it is most abundant in *D. polylepis* and *C. cerastes* venoms (Figure 11). *N*^6^-Methyladenine has been reported to depress cholinergic neurotransmission [170].

2′-Deoxyadenosine was the least abundant purine that we identified; however, its highest concentrations were found in *D. polylepis* and *C. cerastes* venoms (Figure 11). 2′-Deoxyadenosine was found to stimulate neurotransmitter release at cholinergic sites [170].

### 2.5. Neurotransmitters

#### 2.5.1. Acetylcholine

The fifth most abundant organic metabolite in these venoms was acetylcholine, again, owing to its high concentration in *Dendroaspis polylepis* venom [106,171]; however, it was present in all others, albeit at 4–5 orders of magnitude lower abundance than in black mamba venom (Figure 2). Mamba venom acetylcholine targets principally vascular muscarinic receptors promoting vasodilation, the nicotinic neuromuscular junction and, probably secondarily, central nicotinic receptors [4]. Mamba venoms employ an excitatory strategy. In addition to containing acetylcholine, they also possess dendrotoxins, which promote acetylcholine release from nicotinic endplates [101,172], and fasciculins, which function as acetylcholinesterase inhibitors [103,104,105]. Cobras (*Naja*, *Ophiophagus*, *Hemachatus*) adopt a paralytic strategy involving postsynaptic nicotinic receptor antagonists and acetylcholinesterase, so acetylcholine would make no sense as a toxin in these venoms.

#### 2.5.2. γ-Aminobutyric Acid

*Bothrops moojeni* venom contained potentially significant concentrations of γ-aminobutyric acid (GABA), an important inhibitory neurotransmitter (Supplementary Table S1). Titers in all other venoms were 1–3 orders of magnitude lower. As noted in the section on 4GBA, the superior cervical ganglion is a part of the autonomic nervous system and is specifically responsible for “fight-or-flight” responses. Binding of GABA to its receptors on the superior cervical ganglion suppresses escape locomotor behavior in rats [40], which would be advantageous to the snake. Whether the concentrations of GABA in *B. moojeni* venom are sufficient to suppress escape in rodent prey, or ground doves, on which this snake also feeds, is an open question.

### 2.6. Amines and Alkaloids

**Figure 14:**
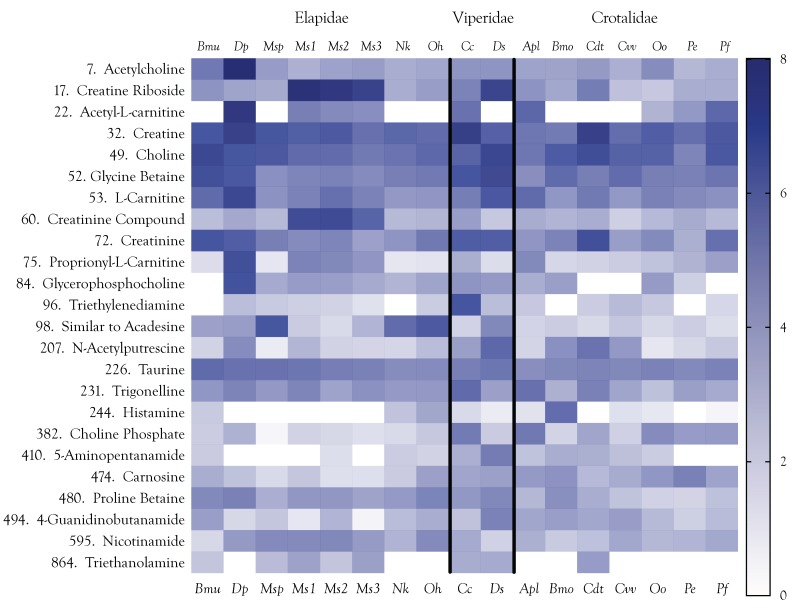
Various amines and cholines (quaternary amines) were identified in the 17 snake venoms. Most were present at low levels, suggesting that functional roles in envenomation are improbable. Others showed modest to high concentrations in specific taxa, but were essentially absent in others. Examples of this pattern include acetyl-l-carnitine, proprionyl-l-carnitine, triethylenediamine, histamine, and 5-aminopentanamide. Taxonomic names: Bmu, *Bungarus multicinctus*; Dp, *Dendroaspis polylepis*; Msp, *Micrurus spixii*; Ms1–3, *Micrurus surinamensis*, 3 individuals; Nk, *Naja kaouthia*; Oh, *Ophiophagus hannah*; Cc, *Cerastes cerastes*; Ds, *Daboia siamensis*; Apl, *Agkistrodon piscivorus leucostoma*; Bmo, *Bothrops moojeni*; Cdt, *Crotalus durissus terrificus*; Cvv, *Crotalus viridis viridis*; Oo, *Ovophis okinavensis*; Pe, *Protobothrops elegans*; Pf, *Protobothrops flavoviridis*.

The venoms examined here contained a variety of amines, many of which are tertiary or quaternary amines (Figure 14). Polyamines, which are among the most significant amines pharmacologically and, perhaps, in terms of abundance as well, were not detected in this study because detection with LC-MS requires derivatization [173]. Derivatization could not be used here as it would have hopelessly complicated identification of other compounds. However, *N*-acetyl-putrescine occurs naturally in venoms, and was detected in potentially significant quantities in the venoms of *D. polylepis*, *D. siamensis*, and *C. d. terrificus*. As a group, amines are interesting because while some of them occur in more or less all venoms examined, several of the most abundant show greatly elevated concentrations specifically in one to several venoms (Figure 14). The most important classes of amines in snake venoms are derivatives of creatine/creatinine, carnitine, and choline.

#### 2.6.1. Creatine and Creatinine

Creatine and analogs, such as cyclocreatine, have antitumor, antiviral, and antidiabetic effects, and protect tissues from hypoxia, ischemia, neurodegeneration, or muscle damage [174], but these effects are difficult to reconcile with functions essential to envenomation. Creatine has a central role in ATP synthesis, where it acts as a phosphate group acceptor to form phosphocreatine. The latter, in turn, acts as a donor of phosphate for conversion of ADP to ATP via the action of creatine kinase (EC 2.7.3.2).

**Figure 15:**
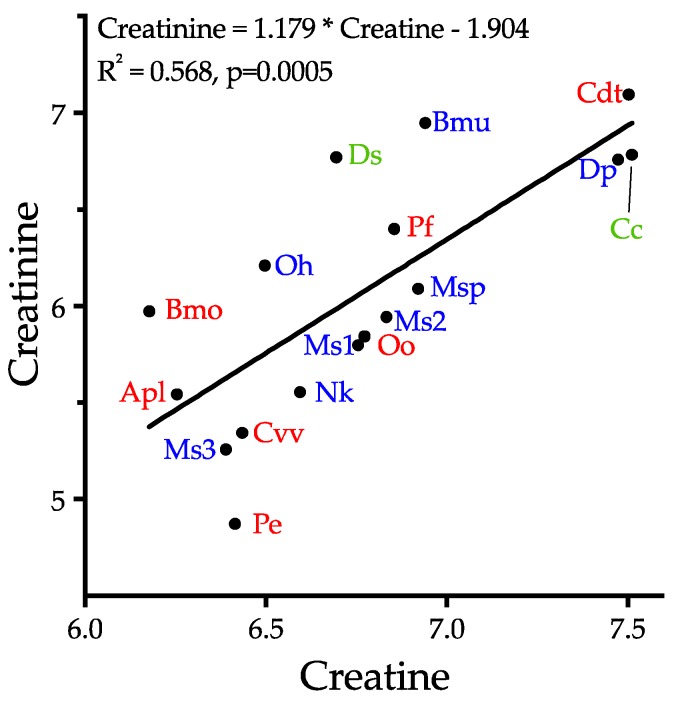
Creatinine levels are well correlated with creatine levels, reflecting their metabolic link; however, they show no obvious relationship to either phylogeny or ecology, suggesting the lack of a functional role in debilitation of prey. It seems reasonable that the elevated levels of these compounds simply reflect the high rate of ATP anabolism and catabolism in the gland, resulting from the demands of protein synthesis. Elapids, red; viperines, green; and crotalines, blue. Taxonomic abbreviations: Apl, *Agkistrodon piscivorus leucostoma*; Bmo, *Bothrops moojeni*; Bmu, *Bungarus multicinctus*; Cc, *Cerastes cerastes*; Cdt, *Crotalus durissus terrificus*; Cvv, *Crotalus viridis viridis*; Ds, *Daboia siamensis*; Dp, *Dendroaspis polylepis*; Mss, *Micrurus spixii spixii*; Ms1–3, *Micrurus surinamensis* 1–3; Nk, *Naja kaouthia*; Oh, *Ophiophagus hannah*; Oo, *Ovophis okinavensis*; Pe, *Protobothrops elegans*; Pf, *Protobothrops flavoviridis*.

Creatinine reportedly has anticonvulsant activity in the CNS [175], but as with the physiological functions of creatine, this appears to be inconsistent with the objectives of snake envenomation. Creatinine is a catabolite of creatine in the arginine metabolic pathway. In the absence of any obvious pharmacology consistent with prey debilitation, we suggest that, like the carnitines, the primary role of creatine is probably to support venom protein synthesis via ATP production, and that the concomitantly high levels of creatinine probably reflect creatine metabolism. While creatinine levels are indeed correlated with creatine levels (*r*^2^ = 0.568, *p* = 0.0005), the two compounds show no apparent relationship with either phylogeny or biology (Figure 15), supporting the hypothesis of a metabolic role, rather than a strategic function. If this hypothesis is correct, the apparent ubiquity of this metabolite (Figure 14) could be taken as support for a non-strategic function.

#### 2.6.2. Carnitines

Like choline, l-carnitine and its derivatives, acetyl-l-carnitine, and propionylcarnitine, all of which are found in some venoms, are also quaternary ammonium compounds. Acetyl-l-carnitine (ALC) is derived from acetylation of carnitine in mitochondria. In addition to transporting long-chain fatty acids to mitochondria for β-oxidation, ALC provides acetyl groups for acetylcholine synthesis, exerts a cholinergic effect, and can be incorporated into glutamate, glutamine, and GABA [176]. Mamba venoms have high levels of both acetylcholine and ALC (Supplementary Table S1). In fact, the *D. polylepis* ALC level exceeds that of most other venoms by 3–4 orders of magnitude, suggesting that its primary function in venoms is as a precursor for acetylcholine. Secondarily, at supraphysiologic concentrations, ALC is neuroprotective in animal models of cerebral ischemia [177,178,179,180]. Neuroprotection is accomplished by suppression of neuronal activity, which would be consonant with snake envenomation strategies [4].

Only the venom of *D. polylepis* showed a relatively high concentration of proprionyl-l-carnitine, although it was also present in the venoms of *M. surinamensis*, *C. cerastes*, *A. p. leucostoma*, and the two *Protobothrops* species (Figure 14). Proprionyl-l-carnitine acts directly upon vascular epithelium to activate endothelial nitric oxide synthase, resulting in the production of nitric oxide, a potent vasodilator [181]. It also counteracts the vasoconstrictor activity of endothelin [182].

#### 2.6.3. Cholines

In addition to acetylcholine, mentioned above, venoms also contain choline itself, and choline phosphate (Supplementary Table S1). Interestingly, choline is most abundant in venoms of *D. siamensis*, *B. multicinctus*, and *C. d. terrificus*, rather than in *D. polylepis*, as might have been anticipated due to its high acetylcholine content. Nor do choline levels mirror those of choline phosphate, which is most abundant in *A. p. leucostoma* venom.

#### 2.6.4. Betaines

Glycine betaine, a trimethyl derivative of glycine, was the sixth most abundant amine in the venoms we examined. Betaine’s role as a methyl donor is well known [183], as is its function as an osmolyte [184,185]. A betaine-GABA transporter, BGT-1, was isolated from dog kidney by Yamauchi et al. [186], and that same year, a highly similar GABA transporter was isolated from mouse brain [187]. Schousboe et al. [188] concluded that BGT-1 receptors govern seizure susceptibility, but what role they play is unclear. Borden and colleagues [189,190] found that BGT-1 is widely distributed throughout the human brain and outside the central nervous system, but in the CNS its distribution is largely astrocytic.

On the basis of plasma and urine concentrations, Lever et al. [191] concluded that glycine betaine, but not proline betaine [192], is important in the biochemistry of humans and other mammals. Snakes do not accumulate compounds in their venoms unless they contribute to prey immobilization or serve a function in the venom gland. If so, then why do all snake venoms examined contain modest to significant amounts of glycine betaine and roughly 12-fold lower levels of proline betaine?

Presently, four classes of GABA transporters have been identified: GAT-1, GAT-3, GAT-4, and GAT-2, which is the same as BGT-1 [186]. BGT-1 also transports glycine betaine in addition to GABA. Matskevitch et al. reported that oocytes expressing BGT-1 were equally depolarized by 1 mM glycine betaine or GABA [193]. Takanaga et al. [194] found that BGT-1 is expressed at the blood–brain barrier (BBB) and participates in GABA transport across the BBB. They found that GABA transport across the BBB was inhibited by 22% using 0.5 mM glycine betaine and Barakat et al. [195] reported that ≥200 µM betaine is a competitive blocker of BGT-1 transporters.

When GABA transporters in neuronal and glial cells are inhibited with nipecotic acid, GABA diffuses from the brain into the bloodstream in rats [196]. Efflux of GABA across the blood–brain barrier may compensate for normal GABA reuptake by neuronal and glial cells [194]. 

It is possible that venom glycine betaine interferes in some way with prey GABA levels. Given that venoms contain various small metabolites that function as GABA agonists and that are also pro-convulsants (2-OA, 4GBA, I4AA), it is possible that betaine also acts as a pro-convulsant. On the other hand, GABA_A_ receptor agonists produce sedation at only 10% occupancy [80]. It may be that inhibitory concentrations of glycine betaine block BGT-1, augmenting local concentrations of GABA, thereby inducing sedation, hypotension, and bradycardia [81].

Proline betaine, also known as stachydrine, most likely has a different function. It has been shown to improve endothelial cell viability and to inhibit cell senescence by modulating p16^INK4A^, a tumor suppressor protein that transduces senescence signals to drive cells into senescence [197,198,199]. In hyperglycaemia, proline betaine counteracts the harmful effects of high blood glucose by downregulating p16^INK4A^ levels and by blocking inhibition of SIRT1. Mercken et al. [200] found that SRT2104, a synthetic small molecule activator of SIRT1, extended both mean and maximal lifespan of mice. It is impossible to draw any firm conclusions at this point but, hypothetically, given its beneficial effects on endothelial cells and its apparent ubiquity in snake venoms (Figure 14), perhaps proline betaine serves to extend the longevity of venom gland epithelial cells.

#### 2.6.5. Taurine

Taurine is technically an amino sulfonic acid, since it lacks a carboxyl group. Nonetheless, it is one of the most abundant amino acids in mammals [201]. It occurs in virtually all tissue types at relatively high concentrations, and impacts a wide variety of biological processes, a number of which are pertinent to envenomation.

Nearly six decades ago, Curtis and Watkins [27] and, later, Curtis et al. [62], reported that iontophoretically applied taurine has a depressant effect on cortical neurons and spinal interneurons. Pasantes-Morales et al. [202] found that, in chicken retina, application of taurine depressed the b-wave of the electroretinogram, a finding confirmed by Bonaventure et al. [203] for intravitreal injections of taurine. They reported that the depressant action of taurine, but not of GABA, was abolished by strychnine, an antagonist of glycine and acetylcholine receptors. Conversely, picrotoxin, an antagonist of GABA_C_ receptors, abolished the depressant action of GABA, but not taurine. These findings were significant because Curtis et al. [204,205] had earlier shown that GABA-like amino acids act presynaptically, and are antagonized by picrotoxin and bicuculline; whereas glycine-like amino acids are blocked by strychnine and act postsynaptically. Bonaventure et al. concluded that both taurine and GABA act as inhibitory neurotransmitters in the retina [203].

Okamoto et al. [206] showed that the hyperpolarizing action of taurine on Purkinje cell dendrites in guinea pig cerebellar slices was selectively and competitively antagonized by 200 µM TAG (6-aminomethyl-3-methyl-4H,1,2,4-benzothiadiazine-1,1-dioxide), an amino acid antagonist, while actions of GABA, glycine and beta-alanine were scarcely affected. TAG reversed the hyperpolarization induced by exogenously applied taurine at the same potential, and 200 µM TAG completely and reversibly blocked the synaptic potential. Okamoto et al. [206] suggested that taurine may be an inhibitory neurotransmitter in stellate neuronal synapses on Purkinje cell dendrites. Lin et al. [207] subsequently proposed that taurine might be used by amacrine cells as a neurotransmitter in rabbit retina. Kamisaki et al. [208] found that addition of 10 µM taurine to the Ca^2+^-free medium perfusing rat cerebral cortical synaptosomes significantly reduced the depolarization-evoked release of Glu, Asp, and GABA. The taurine-induced reduction in GABA release was attenuated by phaclofen, a GABA_B_ antagonist, but not by bicuculline, a GABA_A_ antagonist. However, these antagonists did not block the effects on Glu and Asp release. Taurine released by neurons appears to suppress further transmitter release in much the same fashion as adenosine [4].

Lombardini [209] reported that taurine stimulates ATP-dependent calcium ion uptake and inhibits protein phosphorylation; however, Foos and Wu [210] found that taurine strongly inhibits ^45^Ca^2+^ influx with no effect on efflux. Under prolonged l-glutamate stimulation, neurons release significant (mM) amounts of taurine, which then acts extracellularly to reduce cytoplasmic Ca^2+^ levels by acting upon both transmembrane ion transporters and intracellular storage pools. Specifically, taurine prevents Na^+^/Ca^2+^ ATPase from operating in reverse mode under exocytotic conditions (whereby the ATPase pumps Ca^2+^ in instead of out). In addition, taurine reduces the production of IP_3_ in both control and glutamate-stimulated cells, which, in turn, blocks the release of Ca^2+^ from intracellular stores [210]. Later, Wu et al. [211] proposed that taurine exerts its neuroprotective effects by inhibiting glutamate-induced calcium influx through L-, P/Q-, N-type voltage-gated calcium channels (VGCCs) and NMDA receptor calcium channels. They further suggested that taurine protects neurons against glutamate excitotoxicity by opening chloride channels to prevent glutamate-induced calcium influx, a mechanism that also has anti-apoptotic effects [212].

Hussy et al. [213] investigated agonist properties of taurine on glycine and GABA_A_ receptors of rat supraoptic magnocellular neurons. They found that responses to 1 mM taurine were blocked by strychnine, but not by gabazine, and were not additive with glycine-induced currents, indicating that glycine receptors were selectively activated. Glycine receptor activation opens Cl^−^-selective channels, and the resulting hyperpolarization prevents the neuron from firing [214]; hence, the inhibitory nature of glycine receptors. Bhattarai et al. [215] found that taurine activates different subtypes of glycine receptors in preoptic hypothalamic area neurons. Moreover, 500 µM taurine activated only glycine receptors, but 3 mM taurine activated both glycine and GABA_A_ receptors. Since glycine receptors regulate the excitability of motor and afferent sensory neurons, including pain fibers, and participate in processing visual and auditory signals [214], agonism of glycine receptors by taurine would be neurosuppressive.

Lastly, El Idrissi et al. [216] found that iv injection of taurine in rats caused hypotension and tachycardia. Taurine significantly reduced systolic, diastolic, and mean arterial blood pressure in freely moving rats. They also found that bath application of taurine to aortic rings caused vasodilation. Thus, injection of exogenous taurine would serve to exacerbate the profound hypotension caused by proteinaceous venom components. Are the quantities of taurine found in snake venoms sufficient to exert a significant pharmacological effect in envenomated prey? At this point, we cannot say; however, its pharmacology is completely consistent with snake envenomation strategies and, if not, it is likely that taurine quantities released from prey tissues are sufficient, after the manner of released adenosine.

#### 2.6.6. Carnosine (β-Alanyl-l-histidine)

We were, at first, puzzled by the absence of histamine from most snake venoms and serotonin from all of the venoms examined in this study (Figure 14, Supplementary Table S1), since many venoms are known to provoke the release of these mediators of the immediate hypersensitivity reaction [217,218,219]. However, low amounts of carnosine reduce blood pressure by acting upon the sympathetic nerve innervating the kidneys, although high concentrations of carnosine had the opposite effect [220,221]. Suppression of mean arterial pressure was blocked by administration of the histamine H3 receptor antagonist, thioperamide [222], whereas augmentation of blood pressure was provoked by high concentrations of carnosine, an effect blocked by the H1 receptor antagonist diphenhydramine, These results exactly parallel the effects of different concentrations of histamine itself, injected intracranially [223], suggesting that carnosine’s suppression of blood pressure is primarily histaminergic. Other studies have suggested that carnosine’s antioxidant properties may underlie its effects on blood pressure, but such effects are probably too slow to be relevant to envenomation.

#### 2.6.7. Lesser Amines and Alkaloids

Numerous studies have reported on the histamine-releasing capabilities of various snake venoms [219,224,225,226,227], based upon their capacity to degranulate mast cells [228,229,230]; however, to the best of our knowledge, histamine has been reported as an actual component of a snake venom only once (*Bitis gabonica*) [231]. Recently, Mamede et al. [232] reported that the inflammatory reaction caused by *Bothrops moojeni* venom is mediated by eicosanoids, histamine, nitric oxide, and bradykinin, principally due to the action of phospholipases, metalloproteases, and serine proteases. We confirm that histamine itself is present at potentially significant levels in *Bothrops moojeni* venom (Figure 14, Supplementary Table S1).

*N*-Acetylhistamine occurs in the venoms of *D. polylepis*, *C. cerastes*, *B. moojeni*, and *C. v. viridis* at low levels, with the highest titer occurring in *Dendroaspis* venom (not shown). When administered ip to mice and rats, *N*-acetylhistamine was found to significantly increase tissue histamine levels, which histidine, a precursor of histamine, did not [233].

The literature appears to be completely silent on the pharmacology of *N*-acetylputrescine, a catabolite of putrescine. Triethylenediamine, trigonelline, 4-guanidinobutanamide, nicotinamide, and triethanolamine are found at very low levels in many or all venoms (Figure 14). In most cases, these may be essentially “housekeeping” levels in glandular tissues and, certainly, no convincing case can be made for a role in envenomation, especially given that methyltyramine has hypertensive effects [234]. 4-Guanidinobutanamide is an intermediate between arginine and 4-guanidinobutanoate, which has been discussed above.

### 2.7. Amino Acids

**Figure 16:**
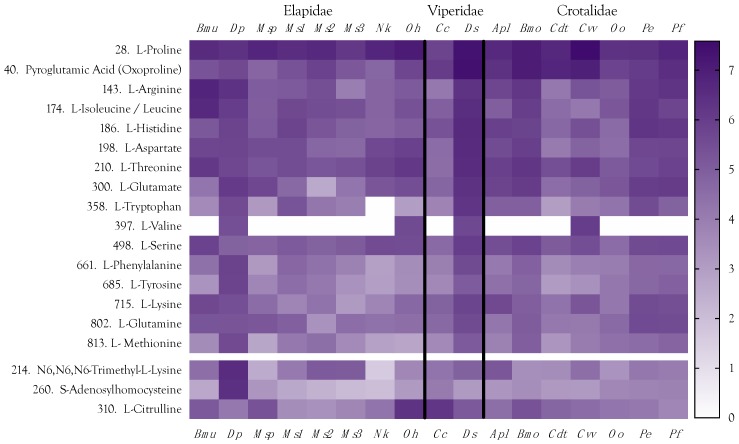
Free amino acids found in venoms, listed in the order of abundance. l-arginine serves as the precursor for nitric oxide; therefore, it potentially promotes hypotension. Proline is a major constituent in hypotensive peptides, hence its abundance in *Crotalus v. viridis* venom. Pyroglutamic acid (oxoproline) likewise blocks the N-terminus of crotaline and viperine hypotensive peptides; hence its greater abundance in those venoms. Trimethyl-lysine is a precursor for carnitine synthesis. Both trimethyl-lysine and proprionyl-l-carnitine are most concentrated in *D. polylepis* venom. High concentrations of some other amino acids are more difficult to explain. Abundance is scaled on the basis of the log_10_ of the total ion concentration. Taxonomic names: Bmu, *Bungarus multicinctus*; Dp, *Dendroaspis polylepis*; Msp, *Micrurus spixii*; Ms1–3, *Micrurus surinamensis*, 3 individuals; Nk, *Naja kaouthia*; Oh, *Ophiophagus hannah*; Cc, *Cerastes cerastes*; Ds, *Daboia siamensis*; Apl, *Agkistrodon piscivorus leucostoma*; Bmo, *Bothrops moojeni*; Cdt, *Crotalus durissus terrificus*; Cvv, *Crotalus viridis viridis*; Oo, *Ovophis okinavensis*; Pe, *Protobothrops elegans*; Pf, *Protobothrops flavoviridis*.

Seventeen of the 20 proteogenic amino acids were found in venoms (Figure 16). Of the remaining three, glycine would not have been detected because of the mass cutoff that was specified in the mass spectrometer. The other two (asparagine, cysteine) were not present at detectable levels, in part because chromatography on the HILIC column was not optimized for separation and detection of all amino acids. In addition to the proteogenic amino acids, several non-proteogenic amino acids were detected. These included *N*-acetyl-l-glutamate, ornithine, l-citrulline, and *N*^6^,*N*^6^,*N*^6^-trimethyl-l-lysine (Figure 16). The latter serves as a precursor in carnitine synthesis [235]. Interestingly, *D. polylepis* venom has much higher levels of both *N*^6^,*N*^6^,*N*^6^-trimethyl-l-lysine and proprionyl-l-carnitine. *N*-Acetyl-l-glutamate is involved in arginine synthesis, but can also be deacetylated by amino acid *N*-acetyltransferase [E.C. 2.3.1.1] to release glutamate. However, it was most abundant in *B. multicinctus* venom, which also had the highest level of arginine, suggesting that arginine synthesis may be its primary function. Citrulline and ornithine are also both intermediates in the arginine cycle; however, these two amino acids do not appear correlated with each other or with arginine (Figure 16). Citrulline was highly abundant in *O. hannah* and *C. cerastes* venoms, while ornithine showed low to modest concentrations in all venoms. We can offer no convincing explanations for the functions of these two compounds.

The most abundant free amino acid in these venoms was L-arginine, which serves as a substrate for nitric oxide synthase (E.C. 1.14.13.39) in the production of nitric oxide, a potent inducer of hypotension (Figure 16). The next most abundant free amino acid is proline, which is a major constituent of hypotensive peptides, including bradykinin-potentiating peptides and structurally related compounds (see the subsequent section on peptides). Not surprisingly, *Crotalus v. viridis* presented the highest proline concentration, followed by *Ophiophagus hannah*. Isoleucine was the third most abundant amino acid, but this is could be a combination of both isoleucine and leucine, since the mass spectrometer cannot distinguish these.

We had expected to find elevated titers of glutamate and/or aspartate in *D. polylepis* venom, since the mambas employ an excitatory envenomation strategy [4], but the levels of these acidic amino acids are not exceptional in any venom. In fact, our pooled sample of *D. polylepis* venom had the lowest level of the four elapid venoms, exceeded by the levels in *D. siamensis* and several crotaline venoms. Moreover, because kraits and cobras employ paralytic envenomation strategies, an excitatory role for these amino acids seems unlikely. On the other hand, a role for glycine might be possible, but as mentioned, we did not gather data in that low a mass range.

### 2.8. Carbohydrates

**Figure 17:**
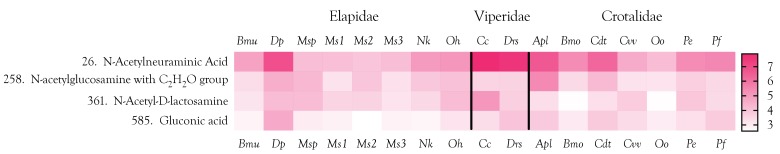
Relatively few mono- and disaccharides were found in these venoms and all were in the *N*-acetylated form. *N*-Acetylneuraminic acid is a common terminating sugar in the branched, asparagine-linked glycan moieties of snake venom glycoproteins. Mannose was not recorded, but it could not have been detected under the conditions were used, except at very high concentrations. Taxonomic names: Bmu, *Bungarus multicinctus*; Dp, *Dendroaspis polylepis*; Msp, *Micrurus spixii*; Ms1–3, *Micrurus surinamensis*, 3 individuals; Nk, *Naja kaouthia*; Oh, *Ophiophagus hannah*; Cc, *Cerastes cerastes*; Ds, *Daboia siamensis*; Apl, *Agkistrodon piscivorus leucostoma*; Bmo, *Bothrops moojeni*; Cdt, *Crotalus durissus terrificus*; Cvv, *Crotalus viridis viridis*; Oo, *Ovophis okinavensis*; Pe, *Protobothrops elegans*; Pf, *Protobothrops flavoviridis*.

Four mono- and disaccharides were identified in the 17 venoms examined. Consistent with reports by Birrel et al. [236] and Zelanis et al. [237], all were *N*-acetylated forms (*N*-acetylneuraminic acid, *N*-acetyl-d-galactosamine-4-sulfate, *N*-acetylglucosamine, bearing a C_2_H_2_O group, and *N*-acetyl-d-lactosamine). *N*-Acetylneuraminic acid and *N*-acetylglucosamine are common terminating sugars of asparagine-linked glycan moieties of venom glycoproteins [237,238,239,240]. *N*-acetylneuraminic acid was relatively abundant in all venoms examined, while *N*-acetylglucosamine was found at higher levels only in the venoms of *C. cerastes*, *B. multicinctus*, and *C. d. terrificus* (Figure 17). In the only such report of which we are aware, Gowda and Davidson [241] found that *Naja kaouthia* venom contains heavily glycosylated high-molecular-weight proteins bearing *N*-acetyl-lactosaminyl oligosaccharides. Our results indicate that this sugar is more common in elapid and viperine venoms than in crotaline venoms. We did not detect mannose (MW = 180.156), which is arguably the most abundant neutral sugar reported in snake venom glycoprotein glycan moieties; however, as a neutral sugar, only very large quantities of mannose could be detected under the LC-MS conditions we used.

### 2.9. Metabolite Biosynthetic Pathways

Aird et al. [173] found that the enzyme, spermine synthase, was strongly upregulated in the venom glands of *Protobothrops mucrosquamatus*, leading to the discovery of polyamines as constituents of snake venoms. However, that case was fortuitous. Generally speaking, we would expect to find upregulation of enzymes in pathways leading to formation of the metabolites reported here, but by virtue of being catalytic, enzymes have no stoichiometry. That is, a massive upregulation may not be required to produce a considerable amount of some metabolites. Enhanced production of small metabolites is normally expected to be more complicated than in the case of spermine synthesis, in that not only should anabolic enzymes be upregulated, but catabolic enzymes may be downregulated or inhibited. Furthermore, for enzymes that are bidirectional, there must be some way of removing and sequestering the product so as to shift the equilibrium to facilitate additional metabolite formation.

### 2.10. Final Considerations about Organic Metabolites

Functional organic components of snake venoms may constitute a more complex case than their proteinaceous counterparts. Venom proteins and peptides represent the endpoints of transcription and translation of genes that are specifically upregulated for that purpose. In contrast, organic metabolites that have been weaponized in venom glands represent intermediates in complex networks of metabolic enzymes. Most of these enzymatic reactions are reversible, and the direction of catalysis is governed by relative concentrations of substrates and products. Moreover, the product of one reaction is the substrate for another. How can any metabolite then become an endpoint? We believe that the only possible answer is sequestration and transport out of venom gland cells into the lumen of the gland, and we are now investigating this matter.

### 2.11. Peptides

**Figure 18:**
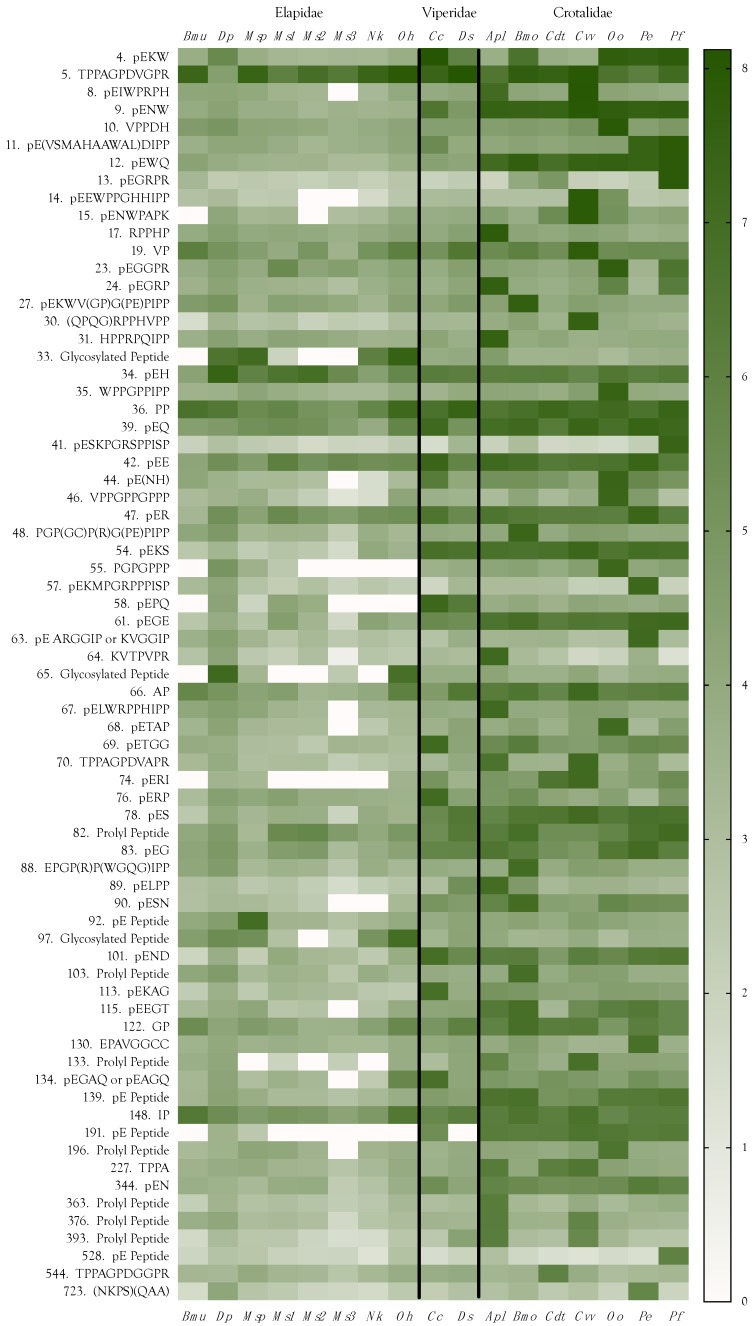
All venoms examined contained peptides. Those sequenced ranged from 172–1716 Da (2–15 amino acids). Many of these were pyroglutamyl and/or prolyl peptides. Their pharmacologies are largely unknown at this point. Taxonomic names: Bmu, *Bungarus multicinctus*; Dp, *Dendroaspis polylepis*; Msp, *Micrurus spixii*; Ms1–3, *Micrurus surinamensis*, 3 individuals; Nk, *Naja kaouthia*; Oh, *Ophiophagus hannah*; Cc, *Cerastes cerastes*; Ds, *Daboia siamensis*; Apl, *Agkistrodon piscivorus leucostoma*; Bmo, *Bothrops moojeni*; Cdt, *Crotalus durissus terrificus*; Cvv, *Crotalus viridis viridis*; Oo, *Ovophis okinavensis*; Pe, *Protobothrops elegans*; Pf, *Protobothrops flavoviridis*.

In general, peptides tend to be more minor components of elapid venoms than of viperine and crotaline venoms (Figure 18, Supplementary Table S2). Sequenced peptides ranged in mass from 172–1716 Da. and included dipeptides, tripeptides, and oligopeptides of up to 13 residues. However, it is apparent that the 71 peptides detected in this study include less abundant oligopeptides of up to about 15 residues (Supplementary Tables S1 and S2). Moreover, we discovered three glycosylated di- and tripeptides, present mostly in elapid venoms, and most abundant in venoms of *O. hannah* and *D. polylepis* (Figure 18, Supplementary Table S2).

#### 2.11.1. Dipeptides

To the best of our knowledge, only one dipeptide has been reported previously in snake venoms, but we identified 13 in the 17 venoms surveyed. Lunow et al. [242] reported that dipeptides containing an aliphatic amino acid in the P1 position and tryptophan in the P2 position are good inhibitors of the C-domain of angiotensin-converting enzyme (ACE), which reduces blood pressure by degrading hypertensive peptides [243,244]. None of the dipeptides we isolated possessed tryptophan in the P2 position, although one of the tripeptides did. Six oligopeptides had tryptophan in the P3 position (Figure 18). Greene et al. [245] noted that the common characteristics of bradykinin-potentiating peptides from *Bothrops jararaca* venom include an N-terminal pyroglutamate residue, a high percentage of proline residues, with proline at the C-terminus. All of the dipeptides we found had either pyroglutamate as the first residue or proline as the second.

##### Prolyl Dipeptides

Five prolyl dipeptides sequenced during this study included four aliphatic prolyl peptides, Val–Pro, Ala–Pro, Gly–Pro, and Ile–Pro, as well as Pro–Pro (Figure 18, Supplementary Table S2). Though it lacks an N-terminus blocked with pyroglutamate, valylproline is, reportedly, a slight inhibitor of ACE (IC_50_ = 420 µM) [246]. More importantly, it inhibits the human peptide transporter, hPepT2 [247], which is expressed in astrocytes, subependymal cells, ependymal cells and epithelial cells of the choroid plexus [248]. hPepT2 is responsible for clearing di- and tripeptides, ACE inhibitors, and other substances from the cerebrospinal fluid [249,250]. Thus, Val–Pro may prevent the clearance of other venom hypotensive peptides, further deepening the hypotension induced by other venom constituents. In the present study, Val–Pro was most abundant in *C. v. viridis* venom (Figure 18).

The literature appears entirely silent on the subject of Ile–Pro and Pro–Pro pharmacology. However, given its structural and physicochemical similarity to Val–Pro, we hypothesize that Ile–Pro also inhibits hPepT2. A tripeptide, AAP, from the venom of *Deinagkistrodon acutus*, has been reported to inhibit platelet aggregation [251]. It may be that both Val–Pro and Ile–Pro share this function. As mentioned above, we also found Ala–Pro. According to PubChem, “This dipeptide has not yet been identified in human tissues or biofluids and so it is classified as an ‘Expected’ metabolite” (https://pubchem.ncbi.nlm.nih.gov/compound/418040). Gly–Pro, like Val–Pro, was also tested for its capacity to inhibit ACE, and yielded an even more tepid result (IC_50_ = 447 µM). There is one additional possible pharmacological action of the aliphatic proline peptides. Hirota et al. [252] reported that in addition to ACE inhibition, the tripeptides VPP and IPP also release NO from vascular endothelial cells, inducing endothelium-dependent relaxation of isolated aortic rings. It remains to be seen whether these dipeptides have similar activity.

##### Pyroglutamyl Dipeptides

A total of 8 pyroglutamyl dipeptides were identified in this study (pEE, pEG, pEH, pEK, pEN, pEQ, pER, pES). pEK, which was reported in the venom of *Bothrops moojeni* by Menin et al. [253], was more abundant in our samples of *C. cerastes* and *P. elegans* venoms than in *B. moojeni* venom (Figure 18, Supplementary Table S2). Even though levels in other venoms were rather modest, this was one of the few peptides that displayed roughly similar titers among all families of snakes. So far as we are aware, nothing is known about the pharmacology of any of these dipeptides; however, they exhibit distinctly taxon-specific distributions. pEH is quite abundant in *D. polylepis* venom, implying that it may contribute to hypotension. Beyond that, it is most abundant in the three *M. surinamensis* venoms.

In the venoms we surveyed, pEE, the only member of this group with an acidic amino acid in the second position, was most abundant in *C. cerastes* and *P. elegans* venoms (Figure 18). The remaining pE dipeptides (pEG, pEK, pEN, pEQ, pER, and pES) were all most abundant in crotaline venoms and, sometimes, also in viperine venoms.

pEG is striking for its lack of large charged, hydrophilic, or aliphatic residues. We can do little more than speculate about its possible function. It is tempting to suggest that it might block glycine receptors. This peptide is most abundant in *P. elegans* venom with ~10× lower concentrations in all other venoms, except for the two rattlesnakes, which had substantially lower concentrations than all other venoms (Figure 18). PubChem reports that the dipeptide, pyroglutamyl–valine, is essentially insoluble in water. All venom pyroglutamyl dipeptides involve hydrophilic amino acids in the second position.

#### 2.11.2. Pyroglutamyl Tripeptides

Eleven venom tripeptides, displaying a wide range of concentrations, were sequenced in this study (pEKW, pENW, pEWQ, pE(NH), pEKS, pEPQ, pEGE, pERI, pERP, pESN, and pEND, in order of decreasing abundance) (Figure 18, Supplementary Table S2). Inadequate fragmentation prevented us from confirming whether the fourth peptide has the sequence pENH or pEHN. Tripeptides were first discovered in venoms more than 50 years ago. Kato et al. [254] found two tripeptides, pEQW and pENW, in the venoms of *Gloydius blomhoffii*, *Crotalus adamanteus*, *Bothrops jararaca*, and *Protobothrops flavoviridis*, which do not potentiate bradykinin, suggesting that they do not inhibit angiotensin-converting enzyme (ACE). They found that *Daboia russellii* venom contained only pENW, while *Naja atra* venom had neither. Francis and Kaiser [255] reported pENW and pEQW in the venom of *Bothrops asper* at concentrations of 1 and 4.5 mM, respectively, and found that they effectively inhibited two venom metalloproteases (80–90% inhibition) at peptide concentrations below 1 mM. Lo [256] reported the tripeptides pEEW and pEDW from the venoms of the pitvipers, *Protobothrops mucrosquamatus* and *Trimeresurus gramineus*, and pEKS was isolated from *Gloydius blomhoffii* venom [257], but it appears that their pharmacologies are unknown. Lou et al. [258] reported that the tripeptide, KNL, from the venom of *Deinagkistrodon acutus*, is able to inhibit a fibrinogenolytic P-I metalloprotease in the same venom. We did not isolate this peptide, or anything structurally similar.

##### pEKW

Yee et al. [259] reported that two tripeptides, pERW and pEKW, from *Daboia russellii* venom, completely inhibited the gelatinolytic and fibrinogenolytic activities of the metalloproteases, RVV-X, and Daborhagin, at a concentration of 5 mM. We did not isolate pERW; however, pEKW was the most abundant peptide in any of these venoms, reaching its highest concentration in *C. cerastes*, *O. okinavensis*, *P. flavoviridis*, *P. elegans*, *B. moojeni*, and *D. polylepis* venoms (Figure 18). Wagstaff et al. [260] reported that the ≤10 kDa portions of *Cerastes cerastes* and *Echis ocellatus* venoms were predominantly comprised of this tripeptide. Huang et al. [261] found three endogenous tripeptides in the venom of *Protobothrops mucrosquamatus* (pEKW, pENW, and pEQW) that inhibited the activities of multiple metalloproteases in the venom. pEKW was also reported as a metalloprotease inhibitor in *Bothrops jararaca* venom [262] and in *Cerastes cerastes* and *Echis ocellatus* venoms [260]. The observation of Lunow et al. [242] that dipeptides with tryptophan in the P2 position inhibit ACE, may also be applicable to tripeptides that bear a C-terminal tryptophan.

##### pENW

This tripeptide was abundant in all pitviper venoms that we surveyed, and it even occurred at modest concentrations in *C. cerastes*, *B. multicinctus*, and *D. polylepis* venoms (Figure 18). Sciani and Pimenta [263] reported that this tripeptide is found as a module in 9% of all bradykinin-potentiating peptides. Munekiyo and Mackessy [264] found it in ten rattlesnake venoms, and reported that it inhibits and stabilizes several of the major metalloproteases in these venoms. Robeva et al. [265] reported that both pENW and pEEW effectively inhibit hemorrhagic toxin e from the venom of *Crotalus atrox*, and suggested the dual function of inhibiting venom metalloproteases during storage in the venom gland. pENW has also been reported to inhibit platelet adhesion and clot retraction in a dose-dependent manner [266], an activity consistent with envenomation strategy to render the blood of prey incoagulable.

##### pEKS

This peptide was first reported from the venom of *Agkistrodon* (*Gloydius*) *halys blomhoffii* by Okada et al. [257]. The authors offered no pharmacology, but speculated that this and other tripeptides might simply be enzymatically released metabolites. Nearly 45 years later, it is apparent that most abundant short peptides are functional venom constituents, and not metabolic accidents. However, we are no closer to understanding the pharmacology of most of them. This peptide, with its blocked N-terminus, its high pI, and two extremely hydrophilic C-terminal amino acids, is especially intriguing. We suspect that the Lys and Ser residues make this peptide an inhibitor of some prey enzyme, receptor, or ion channel.

##### pEPQ, pEGE, pERI, pERP, pE(NH), pESN, and pEND

An additional seven tripeptides, pEPQ, pEGE, pERI, pERP, pE(NH), pESN, and pEND, were found in these venoms. As mentioned above, we were unable to determine the exact sequence of the peptide pE(NH). However, at this point, it probably does not matter. We are unaware of any reports of these tripeptides in the toxinological literature, much less pharmacological profiles for them. With its C-terminal Pro–Arg sequence, pEPR resembles a shortened version of oligopeptides with 5 or 11 residues. Pyroglutamyl tripeptides may represent hypotensive peptides with only two modules, following the proposal of Sciani and Pimenta [263].

#### 2.11.3. Tetrapeptides

The present study also discovered eight tetrapeptides (pEGRP, pETAP, pETGG, pELPP, pEKAG, pEEGT, pE(GA)Q, and TPPA) (Figure 18, Supplementary Table S2). Orosz et al. [267] reported the µ-opioid receptor activity of synthetic tetra- and pentapeptides having amidated C-termini and blocked N-termini; however, with several aromatic residues and no prolines, their peptides do not resemble the peptides reported here. Except for the last of these tetrapeptides, all appear to be previously unknown. Given their blocked N-termini and/or proline residues, they are likely to be hypotensive in some fashion, though not necessarily ACE inhibitors.

The sequence TPPA is the N-terminal tetrapeptide of an 11-amino acid peptide originally discovered in 1986 by Aird and Kaiser in the venom of *Crotalus v. viridis* (discussed below), and later found in other pitviper venoms [268]. It is likely that the tetrapeptide is simply a degradation product of the full-length peptide, since its distribution parallels those of the two 11-mer isoforms, but it could conceivably have its own biological activity, after the manner of crotalphine and crotoxin [269,270].

#### 2.11.4. Longer Oligopeptides

Longer oligopeptides were about equally divided between those bearing an N-terminal pyroglutamate residue and those bearing paired prolines in the second and third positions (Supplementary Table S2). This latter motif protects the N-terminus from degradation by proline peptidase. Both groups tend to be proline-rich at the C-terminus, as well. Motifs such as -HIPP, -PIPP, -PPIPP, and even -PPP are typical (Supplementary Table S2). While many nominal bradykinin-potentiating peptides (BPPs) have ACE inhibitory activity to one degree or another, many have other activities as well and, in some cases, ACE inhibition is not the primary effect. Gomes et al. [271] found that an N-terminal pyroglutamate residue and high proline content, even with a C-terminal IPP motif, are insufficient to specify bradykinin-potentiating activity.

Ianzer et al. [272] investigated the mechanisms of action of BPP 7a and BPP 10c from *Bothrops jararaca* venom. BPP 7a does not potentiate the effects of bradykinin, and is a weak inhibitor of the ACE C site (40,000 nM). By contrast, BPP 10c is a potent bradykinin potentiator and a potent blocker of the ACE C site (0.5 nM) [272], but both cause persistent hypotension. Silva et al. [273] reported that, in the presence of a saturating concentration of captopril, originally modeled after a *Bothrops jararaca* pentapeptide, BPP 10c, a decapeptide [274], maintained high renal concentrations for over 3 h, confirming its affinity for some renal target and also showing that ACE was not its only target. Camargo et al. [275] reported that a BPP from *Bothrops jararaca* venom activates argininosuccinate synthetase, resulting in increased nitric oxide production.

#### 2.11.5. Oligopeptides

Bradycardia promoted by BPPs is unrelated to their bradykinin-potentiating activities, since bradykinin promotes tachycardia [276]. Some BPPs exhibit high bradykinin-potentiating activity while causing little inhibition of ACE [277]. BPP-5a, from *Bothrops jararaca* venom, induces protracted reductions in mean arterial pressure and heart rate, via a nitric oxide-dependent mechanism that does not involve ACE inhibition [278]. Cardiovascular effects may be of central origin since Bj-PRO-10c is able to cross the blood–brain barrier [273] and central injections, thereof, were able to reduce blood pressure in spontaneously hypertensive rats [273,279]. As mentioned above, it accomplishes this by activating arginosuccinate synthetase [275].

##### pEGRPR

This pentapeptide appears to have been unreported, so far as we have been able to determine. It possesses the C-terminal Pro–Arg sequence noted above for a tripeptide, and for blomhotin (*Gloydius blomhoffii*) [280].

##### pESKPGRSPPISP

This bradykinin-potentiating peptide has been reported repeatedly from *Protobothrops* and *Trimeresurus* venoms [14,15,271,281]. Our sample of *P. flavoviridis* venom had a very high titer of this peptide. Traces were possibly present in *B. moojeni* and *C. cerastes* venoms. Most other venoms were essentially devoid of this constituent.

##### TPPAGPDVGPR

This peptide was first discovered by Aird and Kaiser in 1986 (unpublished), who used Edman degradation to determine the sequences of various low-molecular-weight constituents in the venom of *Crotalus v. viridis*. That venom also showed the highest concentration of this peptide in the venoms examined in the present study (Figure 18). When investigating the BPP-CNP gene of *Crotalus durissus collilineatus*, Higuchi et al. [282] encountered a slight variant of this sequence (TPPAGPDGGPR). As a result, they also re-isolated the *C. v. viridis* peptide, confirming the Val residue in position 8, synthesized the *Cdc* variant, and then subjected both peptides to exhaustive cardiovascular pharmacological testing. The genetic and chromatographic data were presented by Shigesada Higuchi at the 14th World Congress of the International Society on Toxinology, held in Adelaide, Australia, in 2003 (Supplementary Table S1). Peptide synthesis and cardiovascular pharmacology studies in collaboration with Saad Lahlou at Universidade Federal de Pernambuco were just being initiated. 

Lahlou determined that in both conscious and anesthetized rats, bolus iv doses of the *Cdc* peptide as large as 600 µg/kg had no discernible effect on mean arterial pressure or heart rate during the test period. In captopril-pretreated rats, it also had no effect on either parameter and, in isolated, perfused rat heart preparations, it caused no change in left ventricular systolic pressure [268]. The *Cvv* peptide likewise induced no changes in mean arterial pressure or heart rate when administered to conscious or anesthetized rats in bolus iv doses up to 600 µg/kg. However, in captopril-pretreated rats, bolus doses of 30, 100, and 300 µg/kg precipitated statistically significant hypotension within 30 s, which persisted throughout the recording period. From its pharmacology, it was clear that this 11-residue peptide does not inhibit ACE, it does not release Arg from its C-terminus for production of NO, and it does not inhibit aminopeptidase P. Instead, its sequence and its pharmacology suggested that it functions as an analog of anaphylatoxin C3a [268]. Most recently, Wang et al. [283] reported that this peptide from *C. v. viridis* inhibits smooth muscle contractility in guinea pig ileum, but potentiates it in rat stomach fundus. They surmised that these contrary activities might come from binding to different receptors (bradykinin-B2 receptors in guinea pig ileum and anaphylatoxin C3a receptors in rat stomach fundus.

Hayashi et al. submitted the BPP-CNP gene sequence from *Crotalus durissus terrificus* venom (AAL09427.1) to NCBI in 2000, but did not publish it until seven years later [271]. However, they did not specifically mention this peptide and may have been unaware of it, since it lacks an N-terminal pyroglutamate residue and paired prolines at the C-terminus, signature characteristics of BPPs [263]. In January, 2005, Soares et al. [284] next published this peptide sequence in the sequence of the BPP-CNP gene from *Lachesis muta* venom, although, like the aforementioned authors, they evidently were not aware of its presence. Pahari et al. also [285] published this sequence from the venom gland transcriptome of the desert massasauga (*Sistrurus catenatus edwardsii*) (B0VXV8.1), but did not specifically mention it in the publication. Despite the fact that this obscure peptide had been repeatedly and understandably overlooked by extremely capable investigators, following Higuchi’s 2003 presentation in Adelaide, in November, 2005, Graham et al. [286] reported the peptide in venoms of *Crotalus v. viridis*, *Lachesis muta*, and *Agkistrodon bilineatus*, concluding that it was a bradykinin-inhibitory peptide. Given the well-documented and manifestly hypotensive nature of crotaline venoms and their peptidyl components [4,287,288,289,290,291], that makes no sense.

With its widespread distribution among pitvipers, and the fact that the *Cvv* peptide accounts for approximately 1% of the crude venom by mass [268], this toxin is unquestionably synergistic, with one or more of the BPPs in these venoms, which probably act at a variety of sites to produce coordinated hypotension. Interestingly, the apparently inactive *Cdc* toxin is expressed at almost undetectable levels, and may represent a defective toxin that is experiencing negative selection [292].

##### pEEWPPCHHIPP

This 11-residue peptide is evidently a BPP, given its blocked N-terminus and paired C-terminal prolines. This sequence is novel, but it is similar to sequences reported previously in venoms of *Lachesis muta* [284], *Crotalus adamanteus* [293], and *Trimeresurus gramineus* [281].

##### (QPGQ)RPPHVPP

This peptide, like the preceding one, is restricted to *C. v. viridis* venom (Figure 18). The sequence of the C-terminal 7 residues is certain. So is the composition of the N-terminal four residues, but due to a lack of subfragmentation, the N-terminus cannot be ordered. From the C-terminal sequence, this is probably a BPP. It is also likely that the P2 residue is proline, which would protect the P1 residue, for which the most likely candidate is glutamine, based on other peptide sequences. P3–P4 could then be either Gln–Gly or Gly–Gln.

##### pENWPAPK

This BPP is most similar to BPPs 7b (pENWPSPK) and 10e (pENWPSPKVPP) from *Bothrops cotiara* venom [294]. Among our samples, this peptide was essentially restricted to *Crotalus v. viridis* venom (Figure 18).

##### pETGG

This is an odd sequence in that while the N-terminus is protected, the remaining three residues are small and/or hydrophilic, without a protective proline. We cannot even hazard a guess as to its pharmacology, but we suspect that it may be pharmacologically different from anything reported to date.

##### pEGRP

This is probably a BPP, given its blocked N-terminus and proline in the P4 position, flanked by arginine. We are not aware that it has been reported previously.

##### RPPHP

This peptide appears to be a BPP fragment. It is identical to the middle portion of BPPs from *Bothrops jararaca* (pEQRPPHPPIPPAP) and *Bothrops fonsecai* (pEARPPHPPIPPAP) venoms; however, it is difficult to imagine an enzymatic mechanism that would cut it at the two points necessary. In addition, in our 50 most abundant compounds, there was no evidence of a parent peptide from which it might have been derived, and it was found only in our pooled sample of *Agkistrodon p. leucostoma* venom (Figure 18). The paired prolines in positions 2 and 3, and the C-terminal proline, suggest that it may be fully functional as a BPP.

##### pEKAG

Like pETGG above, this is a peculiar tetrapeptide sequence. Again, the N-terminus is blocked and the Lys–Ala–Gly sequence is hydrophilic and unprotected with Pro, although this peptide is basic.

##### pELPP

Given its blocked N-terminus and proline-bearing C-terminus, this is probably a very hydrophobic, hypotensive tetrapeptide, but if ACE is not its target, it is hard to say what might be.

##### EPAVGGCC

This peptide appeared in *Protobothrops elegans* venom at a very high concentration and, possibly, in *Dendroaspis polylepis* venom at a low concentration. All other venoms were essentially negative (Figure 18). A BLAST search of the NCBI database yielded no credible matches, so it is not a degradation product of any known protein. With paired cysteines at the C-terminus, this does not appear to be a hypotensive peptide, although it does have a proline in the second position. We cannot offer a hypothetical explanation of its pharmacological role in envenomation, if it has one.

##### Final Considerations about Peptides

We did not detect any homologs of the poly-His–poly-Gly metalloprotease-inhibitory peptide isolated from *Echis ocellatus* venom by Wagstaff et al. [260]. Overall, elapids appear to make far less use of short peptides (Figure 18), although some peptides (QEW, pENW, pEK, pEKS) appear to be widely distributed across most taxa. Crotalines generally appear to make the greatest use of peptides, although some species do so much more than others. *Crotalus v. viridis*, *P. elegans*, and *P. flavoviridis* all have high concentrations of 7–10 of the most abundant peptides. *Crotalus d. terrificus*, which specializes in crotoxin homologs [295], makes much less use of them. Likewise, *C. cerastes* employs high concentrations of a variety of peptides, while *D. siamensis* does not (Figure 18).

### 2.12. Venom Similarities in Terms of Metabolite and Peptide Content

The 17 venoms examined differed greatly in complexity and composition. For instance, *Dendroaspis polylepis* was the simplest venom in terms of its metabolite composition, owing to its purine intensive strategy. Seven constituents, including four purines, accounted for over 60% of the metabolites in the venom. Adenosine alone comprised 26% of all metabolites and adenine added another 11.6% (Supplementary Table S3). Purines were generally important in elapid and viperine venoms, but not in crotaline venoms (Supplementary Table S3) [12].

In *Bothrops moojeni* venom, 25 constituents comprised the top 60% (Supplementary Table S3). In *Ophiophagus hannah* venom, the most major organic component was 4-guanidinobutyric acid, which represented 17.5% of venom metabolites. It was also the major metabolite of *Bungarus multicinctus* venom, in which it comprised 7.3%. It was the third most abundant compound in *Micrurus spixii* venom, and also made the top 60% in two of three *Micrurus surinamensis* venoms, and in both viper and four pitviper venoms (Supplementary Table S3). 

Citric acid was the dominant component in seven viperid venoms and in that of *Naja kaouthia*, evidently reflecting a greater need to inactivate venom metalloproteases in those venoms. Peptides figured prominently in most crotaline venoms, comprising 48% or all metabolites in *Ovophis okinavensis* venom. Peptides dominated *Crotalus viridis* and both *Protobothrops* venoms. They were also very important in *Cerastes cerastes* venom, where pEKW was the major constituent; however, *Daboia siamensis* adopts a largely non-peptidyl strategy. Choline was in the top 60% of all metabolites in nine venoms.

**Figure 19:**
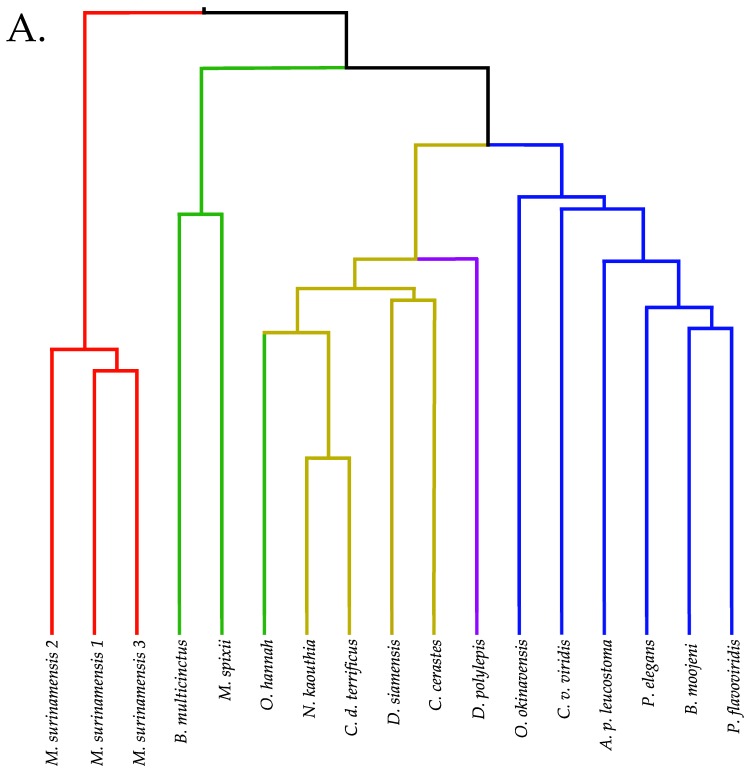

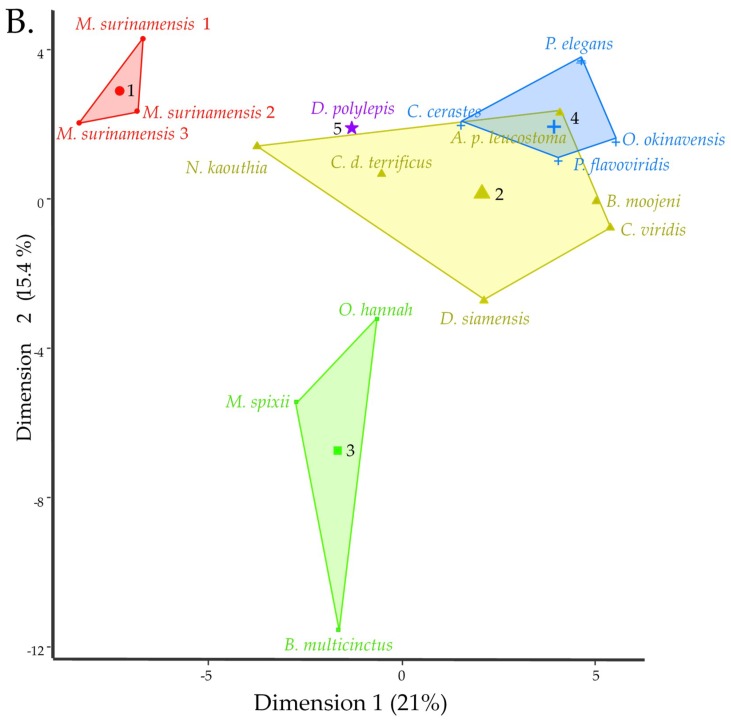
Clustering of 17 venomous snake taxa according to identities and concentrations of the top 100 small metabolites in their venoms. (**A**) Dendrogram of the 17 specimens based upon similarities in their small metabolites and peptides. (**B**) Cluster analysis. Owing to their conspecificity and their preferred fish diet, three specimens of *Micrurus surinamensis* cluster tightly, well separated from everything else (Cluster 1). Cluster 2 comprises New World pitvipers, except for the viperine, *Daboia siamensis*, and *Naja kaouthia*, which is something of a dietary generalist, adults of which feed mostly on rodents and other small mammals. *Micrurus spixii*, which feeds upon snakes and amphisbaenians, most resembles the Ophiophagus Asian taxon, *Bungarus multicinctus*, in terms of venom metabolites. *Ophiophagus hannah* preys upon both snakes and mammals, and also falls into Cluster 3, although in the dendrogram, it clusters with *N. kaouthia*, *C. d. terrificus*, and the two viperines. The reason for this apparent discrepancy is that in the cluster analysis, only the first two of three dimensions can be shown. Cluster 4 includes Asian pitvipers and *Cerastes cerastes*, which sits on the border of Cluster 2. Cluster 5 contains *Dendroaspis polylepis* alone, the venom small metabolome of which is as unusual as its proteome. Colors in the dendrogram (**A**) reflect colors in the cluster analysis (**B**). The R script used to generate the dendrogram and to perform the cluster analysis is provided in Supplementary File 1.

We used the factoextra package with the function fviz_cluster in R to analyze compositional affinities among the 17 venoms studied, based upon the 100 most abundant metabolites. The results were largely as anticipated, but there were some surprises. The three *M. surinamensis* venoms formed a discrete cluster (Cluster 1) separate from everything else, both by virtue of being conspecific and due to this coral snake’s specialization upon fish, especially weakly electric fish (e.g., *Gymnotus carapo*) (Figure 19A,B). Cluster 3 comprised three Ophiophagus taxa, *M. spixii*, *B. multicinctus*, and *O. hannah*. Of these, *O. hannah* showed the greatest affinity for Cluster 2 (Figure 19B), which included New World crotalines and a large viperine, *D. siamensis*. This is most likely because of the king cobra’s predilection for rodents and other small mammals. Cluster 2 also included *N. kaouthia*, an elapid that is something of a dietary generalist, though adults feed largely on mammals. Within Cluster 2, *C. d. terrificus* was well separated from other New World crotalines. *Daboia siamensis* shows the greatest affinity for Ophiophagus elapids. Cluster 4 embraced Old World crotalines (*Protobothrops* sp., and *Ovophis*, plus *Cerastes cerastes*. While belonging to Cluster 2, *A. p. leucostoma* fell squarely in the midst of Cluster 4 (Figure 19B), with which Cluster 2 is closely allied. This is reasonable in that both clusters consist mainly of crotalines. Lastly, *D. polylepis* formed its own cluster (5), due largely to its high titers of purine nucleosides and acetylcholine.

## 3. Materials and Methods 

### 3.1. Venoms

Lyophilized venoms were reconstituted to 250 µg/µL by dissolving them in ultrapure water. Seventeen venoms examined in this study represented the families Elapidae, Viperinae, and Crotalinae (Supplementary Table S4).

### 3.2. Initial Sample Preparation Using Ultrafiltration

Methanol and acetonitrile were purchased from ThermoFisher Scientific, Waltham, MA, USA, and HEPES and PIPES were obtained from Dojindo, Tokyo, Japan. Centrifugal filters were manufactured by Pall Corp., New York, NY, USA.

For each venom, 4 µL of a solution 250 µg/µL (~1 mg) was dissolved in 46 µL of ultrapure water. This solution was immediately mixed with 200 µL of methanol/water to give a final concentration of 50% methanol containing 100 nM HEPES/PIPES. This mixture was transferred to a 10 kDa centrifugal filtration device at 4 °C, and was centrifuged at 13,000 rpm for 10 min. Flow-through amounted to ~230 µL, and was evaporated to dryness. Extracted metabolites were resuspended in 40 µL of 50% acetonitrile/water. However, after the initial experiments were completed, we decided to add several other venoms to the study. The newly added venoms proved to be heavily contaminated with polyethylene glycols. After many experiments to isolate the source of contamination, we discovered that a new batch of Pall filters was responsible. Once the source of the problem was identified, all experiments had to be repeated with a different batch of filters from the same manufacturer. The problem did not recur. However, we now prewash all filters with 400 µL of ultrapure water to wash out contaminants.

Proteomic studies have greatly benefited from the use of molecular cutoff centrifugal filters to reduce sample handling time when buffer exchange is required. Filtration is much faster than with traditional dialysis methods, and sample loss is reduced. Use of these filters allows removal of small molecules from samples, retaining larger molecules on the filter. In a reversal of the usual procedures, we used centrifugal filtration to deproteinate the flow through so as to collect small peptides and metabolites for MS analysis.

Metabolites and peptides collected using methanol extraction all have high polarities, making them difficult to retain on the traditionally used reverse phase columns. In our experience, HILIC columns comprised of zwitterionic materials, have a much broader separation spectrum for mildly to highly polar compounds.

### 3.3. Mass Spectrometry

Samples (3 µL) representing the metabolite and peptide equivalent of 69 µg of each crude venom, were injected into a Thermo Q-Exactive HF mass spectrometer (ThermoFisher Scientific, Waltham, MA, USA) using a Dionex UHPLC Ultimate 3000 liquid chromatograph (Dionex, Sunnyvale, CA, USA). Both hydrophobic interaction and reverse phase chromatography were used to separate metabolites prior to their injection into the mass spectrometer.

#### 3.3.1. Hydrophobic Interaction Chromatography

A SeQuant ZIC-pHILIC HPLC 2.1 × 150 mm column (Merck Millipore, Burlington, MA, USA) was used for separation, flow rate 250 µL/min, using acetonitrile as solvent A and 10 mM ammonium carbonate, 0.2% ammonium hydroxide (pH 9.4) as solvent B. Separation was done in HILIC mode, with a linear gradient from 20% to 80% solvent B in 15 min, followed by a wash for 20 min with 20% acetonitrile, 0.5 M ammonium carbonate in water (solvent C) and, finally, column re-equilibration under starting conditions for 15 min [296].

#### 3.3.2. Reverse Phase Chromatography

A Waters ACQUITY CSH C18 2.1 × 150 mm column (Waters Corporation, Milford, MA, USA) was used for separation, flow rate 200 µL/min, using 0.1% formic acid in water as solvent A, and 0.1% formic acid in acetonitrile as solvent B. Separation was done in reverse phase, with a linear gradient from 1% to 100% solvent B in 20 min, followed by a 5 min wash with 100% solvent B, and column re-equilibration at 1% B for 10 min.

#### 3.3.3. Mass Spectrometry Parameters

The mass spectrometer was operated in both positive and negative ion modes, acquiring one spectrum at MS1 level and MS2 of the 10 most intense peaks in positive mode, switching to negative mode and collecting same spectra. Mass spec settings were as follows, mass range from 70 to 1000 *m/z*, mass resolution 60,000 with Automatic Gain Control at 1 × 10^6^, maximum injection time 30 ms, profile mode for MS1 level. Fixed starting mass at 50 *m/z*, mass resolution 15,000, with AGC at 1 × 10^5^, maximum injection time 50 ms, top 10 most intense ions for MS2 level. Ionization settings were sheath gas flow rate 40, auxiliary gas flow rate 5, spray voltage 3 kV (both modes), capillary temperature 320 °C, and S-lens RF level 60. Retention time, alignment, and peak intensity normalization among samples were performed using 100 nM HEPES/PIPES as internal standards.

#### 3.3.4. Identification and Quantification of Metabolites and Peptides

Compounds were identified using an in-house database of >600 commercial standards, analyzed using the same conditions as in this study. Based upon retention time, precursor ion mass in positive and/or negative mode, and MS2 fragmentation of standards we assigned identifications. Compounds not included in this database were resolved using precursor ion mass and MS2 fragmentation with Compound Discoverer 2.0 (ThermoFisher Scientific, Waltham, MA, USA, 2015) and MzMine 2.3 software [297] with the NIST, Pubchem, Human Metabolome, and KEGG chemical database libraries. In case of ambiguities, in silico fragmentation of suspected compounds or their structural components was performed using the MetFrag Web Tool (https://msbi.ipb-halle.de/MetFragBeta/). Compound names or tentative identifications reflect our best understanding of these structures based upon our interpretations of their fragmentation spectra. Normalization of peak areas was done in Compound Discoverer 2.0.

With regard to peptide sequences, all spectra annotated as peptides were analyzed manually and confirmed by isotopic simulation of parental ions, and their fragment ions using 10 ppm deviation tolerance for a high level of confidence. PEAKs Studio software, version 7 (Bioinformatics Solutions Inc., Waterloo, ON, Canada) was used for de novo sequencing to confirm manual sequence annotation. We are working to create a fragmentation library of these very small venom peptides, which are not available in public fragmentation libraries. This we intend to publish separately when it reaches a greater degree of completeness.

#### 3.3.5. Clustering and Hierarchical Classification

We used the factoextra package in R with the function fviz_cluster to assess affinities of the 17 venoms based upon the identities and quantities of small metabolites and peptides (https://CRAN.R-project.org/package=factoextra). Statistical analysis of metabolite lists obtained for all samples was performed with R packages, initial analysis included almost 900 metabolites, but just the 50 most abundant ones were used for cluster analysis. The script for this analysis is provided in Supplementary File 1.

## Figures and Tables

**Table 1:** Excitatory amino acids and their inhibitory α-decarboxylated derivatives, as determined on rat spinal neurons [27]. Relative potency of the metabolite in question is indicated by the number of + or − symbols. Most importantly, Curtis and Watkins [27] reported that when the α-carboxyl group of the excitatory acidic amino acids is removed (lacking), the pharmacological action was completely reversed, not merely diminished or abolished. Several of each were identified in venoms in this study. Taurine, an aminosulfonic acid, is one of the most abundant amino acids in animal cells, and is a significant constituent of all venoms investigated herein, particularly that of *Bungarus multicinctus*. See Figure 13.

Acidic Amino Acid	Excitation	α-Decarboxylation Product	Inhibition
Aspartic Acid	_+__+__+_	β-Alanine	− − −
Glutamic Acid	_+__+__+_	GABA	− − −
Cysteic Acid	_+__+__+_	Taurine	− − −
β-Hydroxyglutamic Acid	_+__+_	γ-Amino-β-hydroxy-*n*-butyric Acid	− −
*N*-Methylaspartic Acid	_+__+_	*N*-Methyl-β-Alanine	− −
Aminomalonic Acid	_+_	Glycine	− −
α-Aminoadipic Acid	_+_	δ-Aminoadipic Acid	− −
α-Aminopimelic Acid	_+_	ε-Aminocaproic Acid	−
*N*,*N*-Dimethylaspartic Acid	_+_	*N*,*N*-Dimethyl-β-Alanine	0
*N*-Methylglutamic Acid	0	*N*-Methyl-γ-Amino-*n*-butyric Acid	0

## Supplementary Materials

The following are available online at http://www.mdpi.com/2072-6651/10/10/392/s1, Table S1: Metabolites detected in 17 snake venoms, Table S2: Most abundant venom constituents in each venom. All venoms examined contained peptides, ranging in length from 2–15 residues. Many were pyroglutamyl peptides and many others contained one or multiple proline residues. Three were glycosylated dipeptides. Peptide sequences or characterizations, masses, elution times, probable amino acid count, and species with the highest concentration of each are provided. Some sequences contain amino acid codes in parentheses, e.g., #11. This indicates that the composition within the parentheses is confirmed, but the exact sequence is not known. Table S3: Comparisons of organic constituent complexity in 17 snake venoms. Organic compounds are listed in decreasing order of abundance for each taxon or individual, showing their relative importance and illustrating differences between samples. In D. polylepis venom, seven compounds comprised over 60% of all small organic constituents, whereas in B. moojeni venom, 25 compounds comprised this portion. Table S4: Venom suppliers and origins of samples, where known. Data for specimens employed in this study. Supplementary File 1: The R script used to create the dendrogram and to perform the cluster analysis, depicted in Figure 19.

## Data Accessibility

Raw data are being deposited in the MetaboLights repository and may be accessed at: https://www.ebi.ac.uk/metabolights/MTBLS755.
