# Supplementary material for: Organic and Peptidyl Constituents of Snake Venoms: The Picture Is Vastly More Complex Than We Imagined
Source: Toxins (Basel). 2018 Sep 26;10(10):392. doi: 10.3390/toxins10100392 (PMC6215107; doi:10.3390/toxins10100392)
Supplement: Supplementary file 1 [file toxins-10-00392-s001.zip › toxins-350679-supple-final/Invertebrate Organic Toxins.pdf]

Scorpion venoms are complex cocktails of biogenic amines, proteins, peptides, mucoproteins, organic salts and neurotoxins that have shown potential therapeutic applications due to their cytotoxic, apoptogenic, immunosuppressive and antiproliferative properties [1]. The organic chemistry of spider venoms is far better known, having first been discovered in 1953, when spermine and trimethylenediamine were reported in the venom of a South American theraphosid [2]. Spermine and other polyamines were subsequently reported from venoms of other theraphosids [3–5]. Kawai and colleagues found that JSTX, a polyamine toxin bearing a β-alanine moiety [6] from *Nephila clavata* venom blocked glutaminergic neurotransmission at insect and crustacean synapses and later on vertebrate brain [7–12]. Grishin and colleagues reported the structure of argiopine from the venom of the araneid spider, *Argiope lobata*, an acylpolyamine covalently linked to an arginine residue [13]. Aryl and alkyl polyamine antagonists of NMDA receptors have subsequently been found in venoms of various other spider families [14].

1. Sarfo-Poku, C.; Eshun, O.; Lee, K. H. Medical application of scorpion venom to breast cancer: A mini-review. *Toxicon* **2016**, *122*, 109–112.
2. Fischer, F. G., and Bohn, H. Poisonous secretions of bird spiders. *Ann. Che . Liebigs* **1953**, *603*, 232–250.
3. Cabbiness, S. G.; Gehrke, C. W.; Kuo, K. C.; Chan, T. K.; Hall, J. E.; Hudiburg, S. A.; Odell, G. V. Polyamines in some tarantula venoms. *Toxicon* **1980**, *18*, 681–683.
4. M Gilbo, C.; Gilbo, C. M.; Coles, N. W. An Investigation of Certain Components of the Venom of the Female Sydney Funnel Web Spider, Atrax Robustus Cambr. *Aust. J. Biol. Sci.* **1964**, *17*, 758.
5. Duffield, P. H.; Duffield, A. M.; Carroll, P. R.; Morgans, D. Analysis of the venom of the Sydney funnel-web spider, Atrax robustus using gas chromatography mass spectrometry. *Biomed. Mass Spectrom.* **1979**, *6*, 105–108.
6. Xiong, X.-F.; Poulsen, M. H.; Hussein, R. A.; Nørager, N. G.; Strømgaard, K. Structure-activity relationship study of spider polyamine toxins as inhibitors of ionotropic glutamate receptors. *ChemMedChem* **2014**, *9*, 2661–2670.
7. Kawai, N.; Niwa, A.; Abe, T. Spider venom contains specific receptor blocker of glutaminergic synapses. *Brain Res.* **1982**, *247*, 169–171.
8. Kawai, N.; Yamagishi, S.; Saito, M.; Furuya, K. Blockade of synaptic transmission in the squid giant synapse by a spider toxin (JSTX). *Brain Res.* **1983**, *278*, 346–349.
9. Kawai, N.; Miwa, A.; Abe, T. Block of glutamate receptors by a spider toxin. *Adv. Biochem. Psychopharmacol.* **1983**, *37*, 221–227.
10. Kawai, N.; Miwa, A.; Saito, M.; Pan-Hou, H. S.; Yoshioka, M. Spider toxin (JSTX) on the glutamate synapse. *J. Physiol. Paris* **1984**, *79*, 228–231.
11. Saito, M.; Kawai, N.; Miwa, A.; Pan-Hou, H.; Yoshioka, M. Spider toxin (JSTX) blocks glutamate synapse in hippocampal pyramidal neurons. *Brain Res.* **1985**, *346*, 397–399.
12. Pan-Hou, H.; Suda, Y.; Sumi, M.; Yoshioka, M.; Kawai, N. A spider toxin (JSTX) inhibits L-glutamate uptake by rat brain synaptosomes. *Brain Res.* **1989**, *476*, 354–357.
13. Grishin, E. V.; Volkova, T. M.; Arsen’ev, A. S.; Reshetova, O. S.; Onoprienko, V. V. [Structural-functional characteristics of argiopine--the ion channel blockers from the spider Argiope lobata venom]. *Bioorg. Khim* **1986**, *12*, 1121–1124.
14. Albensi, B. C.; Alasti, N.; Mueller, A. L. Long-term potentiation in the presence of NMDA receptor antagonist arylalkylamine spider toxins. *J. Neurosci. Res.* **2000**, *62*, 177–185.
