# Supplementary material for: Organic and Peptidyl Constituents of Snake Venoms: The Picture Is Vastly More Complex Than We Imagined
Source: Toxins (Basel). 2018 Sep 26;10(10):392. doi: 10.3390/toxins10100392 (PMC6215107; doi:10.3390/toxins10100392)
Supplement: Supplementary file 1 [file toxins-10-00392-s001.zip › toxins-350679-supple-final/Table S3.pdf]

Percentile Cutoffs

	Bungarus multicinctus	*Dendroaspis polylepis*	*Micrurus spixii*	*Micrurus surinamensis 1*	*Micrurus surinamensis 2*	*Micrurus surinamensis 3*	*Naja kaouthia*	*Ophiophagus hannah*	*Cerastes cerastes*	*Daboia siamensis*	*Agkistrodon piscivorus leucostoma*	*Bothrops moojeni*	*Crotalus durissus terrificus*	*Crotalus viridis viridis*	*Ovophis okinavensis*	*Protobothrops elegans*	*Protobothrops flavoviridis*
100%	2.770E+08	1.256E+09	4.228E+08	2.710E+08	2.745E+08	1.130E+08	1.951E+08	4.908E+08	7.507E+08	1.011E+09	8.094E+08	8.391E+08	6.012E+08	1.279E+09	7.869E+08	6.815E+08	9.270E+08
10%	2.770E+07	1.256E+08	4.228E+07	2.710E+07	2.745E+07	1.130E+07	1.951E+07	4.908E+07	7.507E+07	1.011E+08	8.094E+07	8.391E+07	6.012E+07	1.279E+08	7.869E+07	6.815E+07	9.270E+07
20%	5.540E+07	2.512E+08	8.456E+07	5.420E+07	5.489E+07	2.260E+07	3.901E+07	9.817E+07	1.501E+08	2.022E+08	1.619E+08	1.678E+08	1.202E+08	2.558E+08	1.574E+08	1.363E+08	1.854E+08
30%	8.310E+07	3.769E+08	1.268E+08	8.129E+07	8.234E+07	3.390E+07	5.852E+07	1.473E+08	2.252E+08	3.032E+08	2.428E+08	2.517E+08	1.803E+08	3.837E+08	2.361E+08	2.045E+08	2.781E+08
40%	1.108E+08	5.025E+08	1.691E+08	1.084E+08	1.098E+08	4.520E+07	7.802E+07	1.963E+08	3.003E+08	4.043E+08	3.238E+08	3.356E+08	2.405E+08	5.116E+08	3.148E+08	2.726E+08	3.708E+08
50%	1.385E+08	6.281E+08	2.114E+08	1.355E+08	1.372E+08	5.649E+07	9.753E+07	2.454E+08	3.754E+08	5.054E+08	4.047E+08	4.195E+08	3.006E+08	6.395E+08	3.935E+08	3.408E+08	4.635E+08
60%	1.662E+08	7.537E+08	2.537E+08	1.626E+08	1.647E+08	6.779E+07	1.170E+08	2.945E+08	4.504E+08	6.065E+08	4.857E+08	5.035E+08	3.607E+08	7.674E+08	4.722E+08	4.089E+08	5.562E+08

ID#	Name	*Bungarus multicinctus*	
		2.770E+08	100.0%
6	4-Guanidinobutyric Acid	2.036E+07	7.3%
49	Choline	1.817E+07	13.9%
38	Imidazoleacetic Acid	1.355E+07	18.8%
23	5-guanidino-2-oxopentanoic Acid	1.292E+07	23.5%
43	Guanine	1.237E+07	27.9%
52	Betaine	1.232E+07	32.4%
21	Guanosine	1.031E+07	36.1%
59	Palmitic Acid	9.539E+06	39.5%
72	Creatinine	8.882E+06	42.7%
32	Creatine	8.705E+06	45.9%
80	Stearic Acid	8.671E+06	49.0%
116	Hydroxystearic Acid	7.041E+06	51.6%
121	Oleic Acid	6.982E+06	54.1%
142	L-Arginine	5.951E+06	56.2%
19	Hypoxanthine	5.754E+06	58.3%
36	PP	4.994E+06	60.1%

ID#	Name	*Dendroaspis polylepis*	
		1.256E+09	100.0%
1	Adenosine	3.271E+08	26.0%
2	Adenine	1.447E+08	37.6%
7	Acetylcholine	1.026E+08	45.7%
3	Citric acid	6.909E+07	51.2%
22	Acetyl-L-carnitine	5.280E+07	55.4%
19	Hypoxanthine	4.227E+07	58.8%
26	Inosine	4.183E+07	62.1%

ID#	Name	*Micrurus spixii spixii*	
		4.228E+08	100.0%
21	Guanosine	5.612E+07	13.3%
23	5-guanidino-2-oxopentanoic Acid	3.004E+07	20.4%
6	4-Guanidinobutyric Acid	2.693E+07	26.7%
43	Guanine	2.305E+07	32.2%
59	Palmitic Acid	1.344E+07	35.4%
33	Glycosylated Peptide = NGP + Hexose -	1.213E+07	38.2%
1	Adenosine	1.092E+07	40.8%
79		1.081E+07	43.4%
92	pE Peptide	9.414E+06	45.6%
80	Stearic Acid	8.433E+06	47.6%
98	Similar to Acadesine	8.375E+06	49.6%
32	Creatine	8.318E+06	51.6%
49	Choline	7.190E+06	53.3%
112		7.161E+06	55.0%
114		7.141E+06	56.6%
144	Prolylleucine	5.781E+06	58.0%
28	Proline	5.518E+06	59.3%
145		5.280E+06	60.6%

ID#	Name	*Micrurus surinamensis 1*	
		2.710E+08	100.0%
17	Creatine Riboside	7.154E+07	26.4%
60	Creatinine Compound	1.434E+07	31.7%
81		1.072E+07	35.7%
93		9.255E+06	39.1%
109		7.424E+06	41.8%
99		7.327E+06	44.5%
23	5-guanidino-2-oxopentanoic Acid	7.265E+06	47.2%
128		6.194E+06	49.5%
32	Creatine	5.671E+06	51.6%
150		5.260E+06	53.5%
34	pEH	3.927E+06	55.0%
201		3.782E+06	56.4%
159		3.297E+06	57.6%
49	Choline	3.161E+06	58.7%
28	Proline	3.108E+06	59.9%
237		3.065E+06	61.0%

ID#	Name	*Micrurus surinamensis 2*	
		2.745E+08	100.0%
17	Creatine Riboside	5.109E+07	18.6%
60	Creatinine Compound	1.626E+07	24.5%
79		9.862E+06	28.1%
99		8.372E+06	31.2%
6	4-Guanidinobutyric Acid	8.177E+06	34.2%
34	pEH	7.677E+06	37.0%
120		6.960E+06	39.5%
112		6.877E+06	42.0%
32	Creatine	6.809E+06	44.5%
131		6.576E+06	46.9%
109		5.015E+06	48.7%
23	5-guanidino-2-oxopentanoic Acid	4.836E+06	50.5%
177		4.276E+06	52.0%
28	Proline	4.171E+06	53.5%
190		3.876E+06	55.0%
203		3.756E+06	56.3%
144	Prolylleucine	3.676E+06	57.7%
180		3.324E+06	58.9%
222		3.223E+06	60.0%
49	Choline	3.070E+06	61.2%

ID#	Name	*Micrurus surinamensis 3*	
		1.130E+08	100.0%
17	Creatine Riboside	2.684E+07	23.8%
23	5-guanidino-2-oxopentanoic Acid	1.048E+07	33.0%
60	Creatinine Compound	4.535E+06	37.0%
3	Citric Acid	3.426E+06	40.1%
79		3.250E+06	43.0%
112		2.915E+06	45.5%
99		2.585E+06	47.8%
32	Creatine	2.452E+06	50.0%
6	4-Guanidinobutyric Acid	2.254E+06	52.0%
177		2.130E+06	53.9%
21	Guanosine	1.901E+06	55.6%
93		1.754E+06	57.1%
49	Choline	1.571E+06	58.5%
59	Palmitic Acid	1.436E+06	59.8%
28	Proline	1.361E+06	61.0%

ID#	Name	*Naja kaouthia*	
		1.951E+08	100.0%
3	Citric Acid	5.898E+07	30.2%
6	4-Guanidinobutyric Acid	2.313E+07	42.1%
23	5-guanidino-2-oxopentanoic Acid	8.979E+06	46.7%
28	Proline	5.562E+06	49.6%
73	Itaconic Acid	4.163E+06	51.7%
21	Guanosine	3.941E+06	53.7%
32	Creatine	3.927E+06	55.7%
225		3.173E+06	57.3%
98	Similar to Acadesine	3.037E+06	58.9%
43	Guanine	2.875E+06	60.4%

ID#	Name	*Ophiophagus hannah*	
		4.908E+08	100.0%
6	4-Guanidinobutyric Acid	8.578E+07	17.5%
33	Glycosylated Peptide = NGP + Hexose -	3.232E+07	24.1%
23	5-guanidino-2-oxopentanoic Acid	3.147E+07	30.5%
3	Citric Acid	2.330E+07	35.2%
21	Guanosine	2.053E+07	39.4%
36	PP	1.652E+07	42.8%
38	Imidazoleacetic Acid	1.589E+07	46.0%
62	Pyridine derivative	1.492E+07	49.0%
43	Guanine	1.396E+07	51.9%
28	Proline	1.213E+07	54.4%
97	Glycosylated Peptide = NG + Hexose +	8.717E+06	56.1%
65	Glycosylated Peptide = NGP + Hexose -	7.352E+06	57.6%
111		7.311E+06	59.1%
98	Similar to Acadesine	6.929E+06	60.5%

ID#	Name	*Cerastes cerastes*	
		7.507E+08	100.0%
4	pEKW	1.327E+08	17.7%
3	Citric Acid	1.103E+08	32.4%
16	N-Acetylneuraminic Acid	7.307E+07	42.1%
32	Creatine	3.245E+07	46.4%
23	5-guanidino-2-oxopentanoic Acid	2.610E+07	49.9%
6	4-Guanidinobutyric Acid	2.277E+07	52.9%
42	pEE	2.232E+07	55.9%
51	pEK	1.841E+07	58.4%
58	pEPQ	1.723E+07	60.7%

ID#	Name	*Daboia siamensis*	
		1.011E+09	100.0%
3	Citric Acid	1.271E+08	12.6%
6	4-Guanidinobutyric Acid	1.189E+08	24.3%
19	Hypoxanthine	5.768E+07	30.0%
16	N-Acetylneuraminic Acid	5.309E+07	35.3%
26	Inosine	3.749E+07	39.0%
36	PP	2.937E+07	41.9%
38	Imidazoleacetic Acid	2.591E+07	44.5%
40	Pyroglutamate (Oxoproline)	2.503E+07	46.9%
21	Guanosine	2.408E+07	49.3%
17	Creatine Riboside	2.403E+07	51.7%
28	Proline	2.255E+07	53.9%
46	Contains Methylpyrimidine	2.065E+07	56.0%
49	Choline	1.952E+07	57.9%
52	Betaine	1.790E+07	59.7%
71		1.265E+07	60.9%

ID#	Name	*Agkistrodon p. leucostoma*	
		8.094E+08	100.0%
3	Citric Acid	1.202E+08	14.8%
18	RPPHP	6.067E+07	22.3%
25	pEGRP	4.299E+07	27.7%
31	HPPRPQIPP	3.436E+07	31.9%
9	pENW	3.154E+07	35.8%
5	TPPAGPDVGPR	2.048E+07	38.3%
23	5-guanidino-2-oxopentanoic Acid	1.663E+07	40.4%
16	N-Acetylneuraminic Acid	1.638E+07	42.4%
42	pEE	1.469E+07	44.2%
64	KVTPVPR	1.431E+07	46.0%
67	pELWRPPHIPP	1.370E+07	47.7%
37	Cytidine	1.359E+07	49.4%
8	pEIWPRPH	1.147E+07	50.8%
12	pEWQ	1.118E+07	52.2%
51	pEK	1.077E+07	53.5%
82	Prolyl Peptide; no pyroglutamate	1.071E+07	54.8%
73	Itaconic Acid	9.986E+06	56.0%
89	pELPP	9.839E+06	57.3%
94		9.168E+06	58.4%
39	pEQ	8.528E+06	59.4%
102		8.256E+06	60.5%

ID#	Name	*Bothrops moojeni*	
		8.391E+08	100.0%
3	Citric Acid	1.011E+08	12.1%
23	5-guanidino-2-oxopentanoic Acid	5.000E+07	18.0%
6	4-Guanidinobutyric Acid	4.951E+07	23.9%
12	pEWQ	4.374E+07	29.1%
27	pEKWV(GP)G(PE)PIPP	4.007E+07	33.9%
9	pENW	2.730E+07	37.2%
48	PGP(GC)P(R)G(PE)PIPP	2.005E+07	39.5%
50	Unknown	1.916E+07	41.8%
39	pEQ	1.378E+07	43.5%
37	Cytidine	1.239E+07	44.9%
73	Itaconic Acid	1.067E+07	46.2%
38	Imidazoleacetic Acid	1.057E+07	47.5%
28	Proline	1.051E+07	48.7%
40	Pyroglutamate (Oxoproline)	1.048E+07	50.0%
88	EPGP(R)P(WGQG)IPP	9.889E+06	51.2%
90	pESN	9.772E+06	52.3%
42	pEE	8.855E+06	53.4%
61	pEGE	8.801E+06	54.4%
103	Prolyl Peptide; no pyroglutamate	8.044E+06	55.4%
78	pES	7.632E+06	56.3%
115	pEEGT	7.091E+06	57.1%
122	GP	7.005E+06	58.0%
36	PP	6.960E+06	58.8%
51	pEK	6.752E+06	59.6%
49	Choline	6.645E+06	60.4%

ID#	Name	*Crotalus d. terrificus*	
		6.012E+08	100.0%
3	Citric Acid	1.397E+08	23.2%
32	Creatine	3.174E+07	28.5%
6	4-Guanidinobutyric Acid	3.064E+07	33.6%
23	5-guanidino-2-oxopentanoic Acid	2.893E+07	38.4%
9	pENW	2.595E+07	42.7%
36	PP	1.782E+07	45.7%
49	Choline	1.350E+07	48.0%
72	Creatinine	1.246E+07	50.0%
73	Itaconic Acid	1.230E+07	52.1%
16	N-Acetylneuraminic Acid	1.019E+07	53.8%
107		7.500E+06	55.0%
108		7.467E+06	56.3%
12	pEWQ	7.375E+06	57.5%
123	Aconitic Acid	6.979E+06	58.6%
125		6.819E+06	59.8%
127		6.745E+06	60.9%

ID#	Name	*Crotalus v. viridis*	
		1.279E+09	100.0%
5	TPPAGPDVGPR	1.234E+08	9.6%
3	Citric Acid	1.157E+08	18.7%
8	pEIWPRPH	9.524E+07	26.1%
9	pENW	8.891E+07	33.1%
6	4-Guanidinobutyric Acid	8.737E+07	39.9%
14	pEEWPPGHHIPP	7.478E+07	45.8%
15	pENWPAPK	7.311E+07	51.5%
20	VP	5.630E+07	55.9%
28	Proline	3.839E+07	58.9%
30	(QPQG)RPPHVPP	3.571E+07	61.7%

ID#	Name	*Ovophis okinavensis*	
		7.869E+08	100.0%
3	Citric Acid	9.365E+07	11.9%
10	VPPDH	8.284E+07	22.4%
4	pEKW	5.127E+07	28.9%
9	pENW	4.890E+07	35.2%
24	pEGGPR	4.481E+07	40.9%
12	pEWQ	3.782E+07	45.7%
35	WPPGPPIPP	2.959E+07	49.4%
44	pE(NH)	2.180E+07	52.2%
45	VPPGPPGPPP	2.099E+07	54.9%
55	PGPGPPP	1.786E+07	57.1%
68	pETAP	1.347E+07	58.8%
36	PP	1.273E+07	60.5%

ID#	Name	*Protobothrops elegans*	
		6.815E+08	100.0%
3	Citric Acid	1.062E+08	15.6%
4	pEKW	3.882E+07	21.3%
9	pENW	3.554E+07	26.5%
12	pEWQ	2.931E+07	30.8%
11	pE(VSMAHAAWAL)DIPP	2.926E+07	35.1%
37	Cytidine	2.760E+07	39.1%
39	pEQ	2.586E+07	42.9%
42	pEE	2.403E+07	46.5%
47	pER	2.033E+07	49.4%
57	pEKMPGRPPPISP	1.775E+07	52.0%
63	pEARGGIP or pEKVGGIP	1.477E+07	54.2%
61	pEGE	1.249E+07	56.0%
83	pEG	1.040E+07	57.6%
73	Itaconic Acid	9.945E+06	59.0%
54	pEKS	8.701E+06	60.3%

ID#	Name	*Protobothrops flavoviridis*	
		9.270E+08	100.0%
3	Citric Acid	1.442E+08	15.6%
11	pE(VSMAHAAWAL)DIPP	7.852E+07	24.0%
12	pEWQ	7.650E+07	32.3%
13	pEGRPR	7.557E+07	40.4%
4	pEKW	6.220E+07	47.1%
9	pENW	4.413E+07	51.9%
41	pESKPGRSPPISP	2.467E+07	54.6%
36	PP	2.355E+07	57.1%
39	pEQ	1.575E+07	58.8%
61	pEGE	1.541E+07	60.5%
23	5-guanidino-2-oxopentanoic Acid	1.228E+07	
6	4-Guanidinobutyric Acid	1.191E+07	
78	pES	1.136E+07	
73	Itaconic Acid	1.099E+07	
37	Cytidine	1.076E+07	
49	Choline	7.751E+06	
50	Unknown	7.444E+06	
110		7.383E+06	
54	pEKS	7.381E+06	
38	Imidazoleacetic Acid	7.174E+06	
32	Creatine	7.149E+06	
117		5.652E+06	
28	Proline	5.624E+06	
